# Microcompartmentalization Controls Silk Feedstock Rheology

**DOI:** 10.1021/acs.langmuir.3c00354

**Published:** 2023-06-21

**Authors:** Marco Elvino Miali, Dror Eliaz, Aleksei Solomonov, Ulyana Shimanovich

**Affiliations:** Department of Molecular Chemistry and Materials Science, Weizmann Institute of Science, Rehovot 7610001, Israel

## Abstract

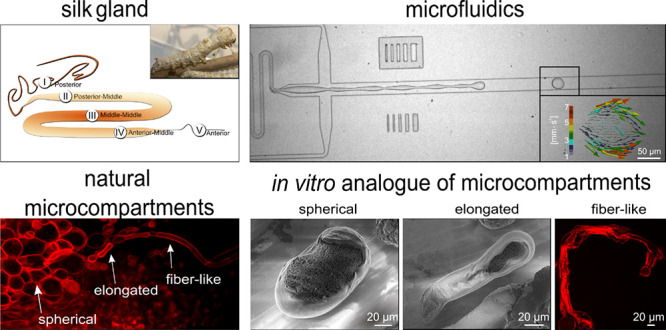

The rheological characteristics of pre-spun native silk protein, which is stored as a viscous pulp inside the silk gland, are the key factors that determine the mechanical performance of the endpoint material: the spun silk fibers. In silkworms and arthropods, microcompartmentalization was shown to play an important regulatory role in storing and stabilizing the aggregation-prone silk and in initiating the fibrillar self-assembly process. However, our current understanding of the mechanism of stabilization of the highly unstable protein pulp in its soluble state inside the microcompartments and of the conditions required for initiating the structural transition in protein inside the microcompartments remains limited. Here, we exploited the power of droplet microfluidics to mimic the silk protein’s microcompartmentalization event; we introduced changes in the chemical environment and analyzed the storage-to-spinning transition as well as the accompanying structural changes in silk fibroin protein, from its native fold into an aggregative β-sheet-rich structure. Through a combination of experimental and computational simulations, we established the conditions under which the structural transition in microcompartmentalized silk protein is initiated, which, in turn, is reflected in changes in the silk-rich fluid behavior. Overall, our study sheds light on the role of the independent parameters of a dynamically changing chemical environment, changes in fluid viscosity, and the shear forces that act to balance silk protein self-assembly, and thus, facilitate new exploratory avenues in the field of biomaterials.

## Introduction

In nature, silk protein-producing animals, including silkworms and spiders, have an exceptional ability to manipulate the self-assembly pathways of a highly unstable protein substrate.^1−3^ Thus, for example, the liquid native silk fibroin (NSF) protein of the *Bombyx mori* (*B. mori*) silkworms, stored inside the silk gland as an aqueous pulp, exhibit typical non-Newtonian behavior when it spins into a crystalline solid fiber.^4−6^ The silk fibroin protein is around ∼400 kDa in size;^7^ it contains GAGAGS repetitive domains that interact during the aggregation process that leads to the formation of either crystalline or amorphous crystal structures, which, in turn, are stabilized via an H-bonded network. As has been shown in previous studies,^8^ silk protein suffers from its high instability and undergoes aggregation^9^ when exposed to slight variations either in pH^10^ or changes in ionic strength (salt concentration)^11^ or under applied shear stress.^12^ Although the effect of pH under bulk conditions has been investigated by several research groups,^13^ the effect of ions, where K and Ca seem to play a major role,^14^ is still poorly understood. Inside the silk gland, the processes are balanced, and even though variations in the chemical environment are present, the structural transitions and macromolecular assembly processes are precisely controlled.^15^ How such control is enabled remains an open scientific question.

The fibroin solution, prior to spinning,^2^ undergoes multiple structural changes that are essential for preparing the viscous pulp for the spinning.^16^ More specifically, the protein’s native fold (the initial random coil conformation of protein as it is produced inside the silk gland) is transformed into a hierarchically ordered β-sheet fold that is further assembled into nanoscale fibrils.^17^ A quite interesting and very important regulatory step in this structural transition is the formation of spherical assembles (hereafter referred to as compartments) that are heterogeneous in size, ranging from 20 nm to 200 μm.^2^ The actual nature of the compartments is not fully understood, neither the structural organization of the protein chains inside the compartments nor the variations in the density of encapsulated fluid. Our recent finding pointed to the dual role played by these compartments.^18^ On the one hand, the formation of the compartments locally decreases the silk-rich solution viscosity (in the posterior-middle part of the silk gland). Thus, it enables the storage of unstable viscous silk pulp in the inner part of the compartment and enables these compartments to flow along the silk gland. On the other hand, the structural transformation of protein^19^ seems to be initiated inside the compartments (in the anterior-middle part of the silk gland), and it is regulated by changes in the environmental conditions, specifically by pH and ionic strength. The microcompartments flow along the gland, at a low shear rate, thus achieving the linear Newtonian fluid behavior of the structured fluid (fluid composed of compartmentalized and bulk silk protein). Within the middle part of the gland, a change in pH is recorded, varying from a typical pH of 7 to pH 5.^20−22^ The anterior part of the gland is characterized by elongational flow (where the lumen cross-section drops from ∼60 to ∼20 μm),^23^ in which the big compartments disassemble and release the cargo of the encapsulated protein-rich solution that, consequently, re-organizes and re-assembles into nanoscale fibrils that are further aligned and spun into microfibers. Even though the events of compartmentalization have been previously reported^2^ and changes in the chemical environment inside the silk gland are also largely known,^24^ there is still a lack of understanding regarding the collective regulatory role of these events during the structural transition of silk protein Interestingly, in our earlier work,^8^ we demonstrated how the volumetric restrictions imposed on native silk fibroin (fibroin protein extracted directly from the silkworm silk gland) dramatically reduce the immediate effect of pH, namely, pH-induced protein aggregation takes place over a longer period of time for compartmentalized protein compared with bulk conditions. A similar effect was observed in the present study.

Here, we utilized a microfluidic droplet platform^8,25−27^ to better understand the link between the formation of the silk protein-rich compartments, changes in the chemical environment, and how these events affect the silk-rich fluid behavior at the microscale level and at the molecular scale as well as how these events modulate the structural transitions in silk protein. To address these challenges, we generated artificial analogue of silk microcompartments by using microfluidic droplet maker devices, from a synthetic analogue of NSF, which is reconstituted silk fibroin (RSF-silk protein obtained via chemical resolubilization of the silkworm cocoons).^28^ We then performed a systematic analysis of the effect of pH, the role of protein concentration, and the contribution of the acting shear forces to changes in the fluid characteristics of silk-rich solution and structural transformations in silk. The environmental conditions that have been used in our experiments are equivalent to those present inside the silk gland. We also built a theoretical model, based on combining the Lattice Boltzmann approach with Ginzburg–Landau free energy functional theory,^29^ to explain and predict how dynamically changing conditions guide the switch in silk fluid characteristics from Newtonian to non-Newtonian behavior (which is relevant to the posterior section of the gland, where silk protein is synthesized and secreted) and how this switch correlates with structural transitions in silk fibroin protein, with its assembly states as well as with the rheology of silk fluid. We anticipate that the above investigated conditions are indeed mimicking the silk gland environment with low precision, in terms of protein concentration and characteristics of the protein-rich fluid. However, our results help to better understand the combined role of compartmentalization, the dynamically changing chemical environment, and the shear forces that act to balance the structure and the self-assembling behavior of silk proteins.

## Results and Discussion

### Microcompartmentalization of Native Silk Protein inside the *B. Mori* Silkworm Silk Gland

**Figure 1:**
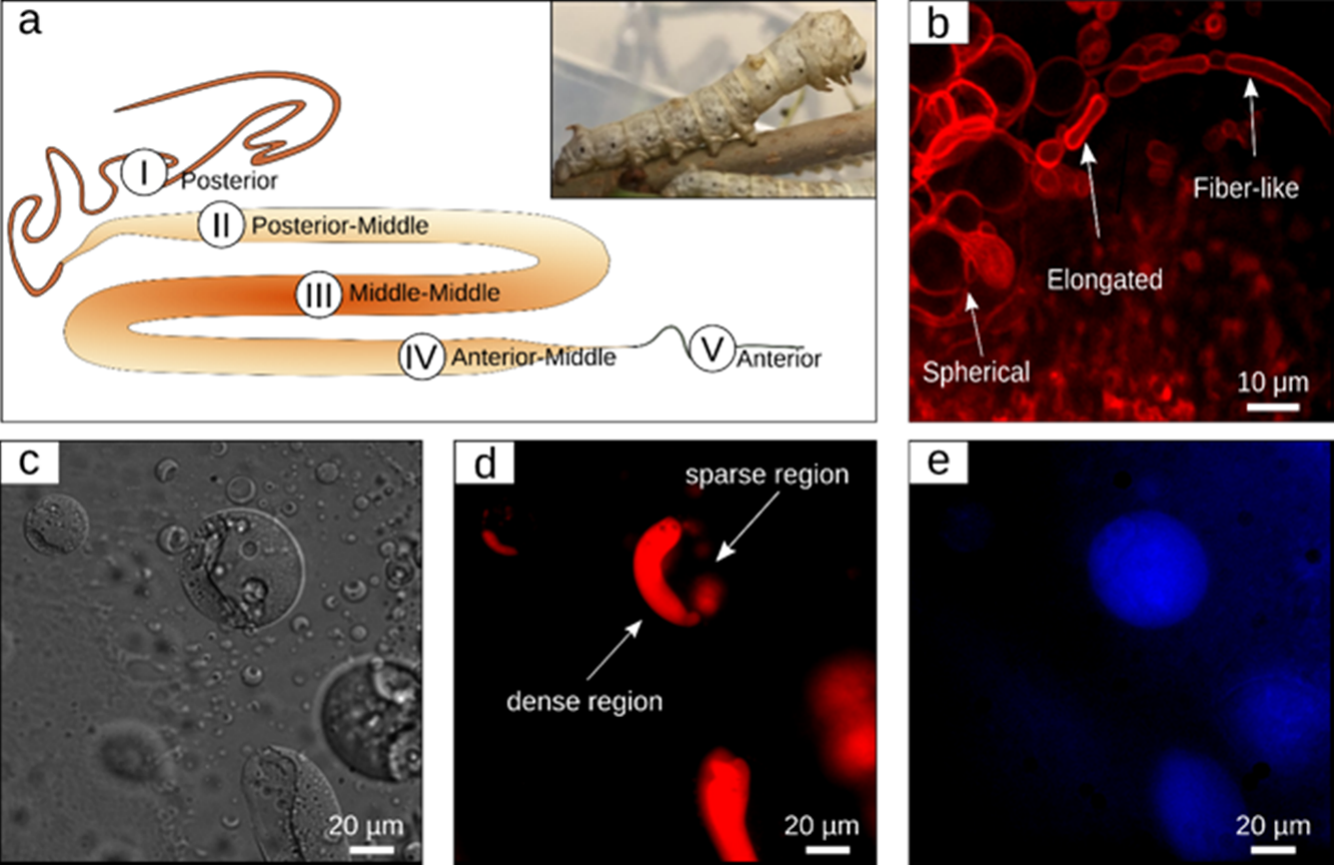
Uniform microcompartment distribution inside silk gland solution and uneven fibroin distribution inside microcompartments (a) silk gland schematics showing the different regions. (b) Fluorescence image inside the silk gland stained with Nile Red showing the microcompartment distribution and shape (spherical, elongated, fiber-like). (c) BF showing microcompartments and fibroin density distribution. (d) Intrinsic fluorescence of the microcompartment revealing the uniform aggregation of fibroin protein. (e) Nile Red staining of the microcompartment showing uneven density of fibroin distribution in static conditions.

In order to better mimic the silk fibroin microcompartmentalization event, which naturally takes place inside the silk gland,^2,8^ we first performed a detailed analysis of the silk protein pulp extracted directly from the silkworm silk gland. To this end, *B. mori* silkworms were grown and dissected at a mature age, i.e., just before they start to spin fibers, following the standard protocol.^30^ The silk gland, containing silk fibroin protein solution, was collected and cleaned with doubly distilled water (DDW). Usually, the highest fibroin protein concentration lacking sericin (glycoprotein coating gum component) is localized at the posterior-middle region of the silk gland, as depicted in Supplementary Figure S1. Therefore, the silk gland is cut at its posterior-middle section for further analysis (see the Experimental Section and Figure 1a and Supplementary Figure S2 and Supplementary Movies S1, S2). Generally, sericin and fibroin phase separate inside and outside the silk gland; thus, granulation in native silk compartments is not due to the presence of sericin. Our optical microscopy analysis revealed that silk protein liquid, from the silkworm’s silk gland, is heterogenous in its nature. More specifically, it contains microcompartments of different sizes and of different shapes, including spherical, elongated (cylindrical), and fiber-like shapes, as shown in Figure 1b. In order to better understand the internal organization of microcompartments, the particles were further visualized using Nile Red staining^31,32^ of the protein’s aqueous content, along with silk protein intrinsic fluorescence (with an excitation of 358 nm and an emission of 460 nm),^33−35^ followed by examination using confocal fluorescence microscopy (Figure 1c–e and Supplementary Figure S3). Although the intrinsic fluorescence of silk provides information about the spatial localization of silk fibroin protein inside the microcompartments, Nile Red staining, due to its solvate-chromic properties, can detect the presence and variations in the polarity of the liquid stored inside the microcompartments. To this end, for Nile Red-based analysis, the polar regions were visualized using confocal microscopy at 554 nm excitation and 638 nm emission. A striking difference in the spatial localization of the fluorescence signals was observed for a single microcompartment particle dispersed in the solution of the silk gland pulp. Whereas the fluorescence emitted from Nile Red is inhomogeneously distributed inside the microcompartment, pointing to the presence of denser (more polar) and less dense regions of protein solution (Figure 1d), intrinsic fluorescence is emitted throughout the entire volume of the particle (Figure 1e). Interestingly, granulation has been observed for the native silk microcompartment (Figure 1c). This peculiar event has been previously studied and characterized by our group^18^ and is associated with the initial stages of silk protein self-assembly. To obtain a better understanding of such complex behavior and to link the dynamically changing chemical environment to the responses of protein-rich fluid, we uncoupled each parameter (the formation of microcompartments, the acting shear forces, variations in pH and in the local protein concentration) and analyzed the consequent changes in fluid behavior and in the protein structure.

### Investigation of the Physico-Chemical Conditions for Silk Compartmentalization

**Figure 2:**
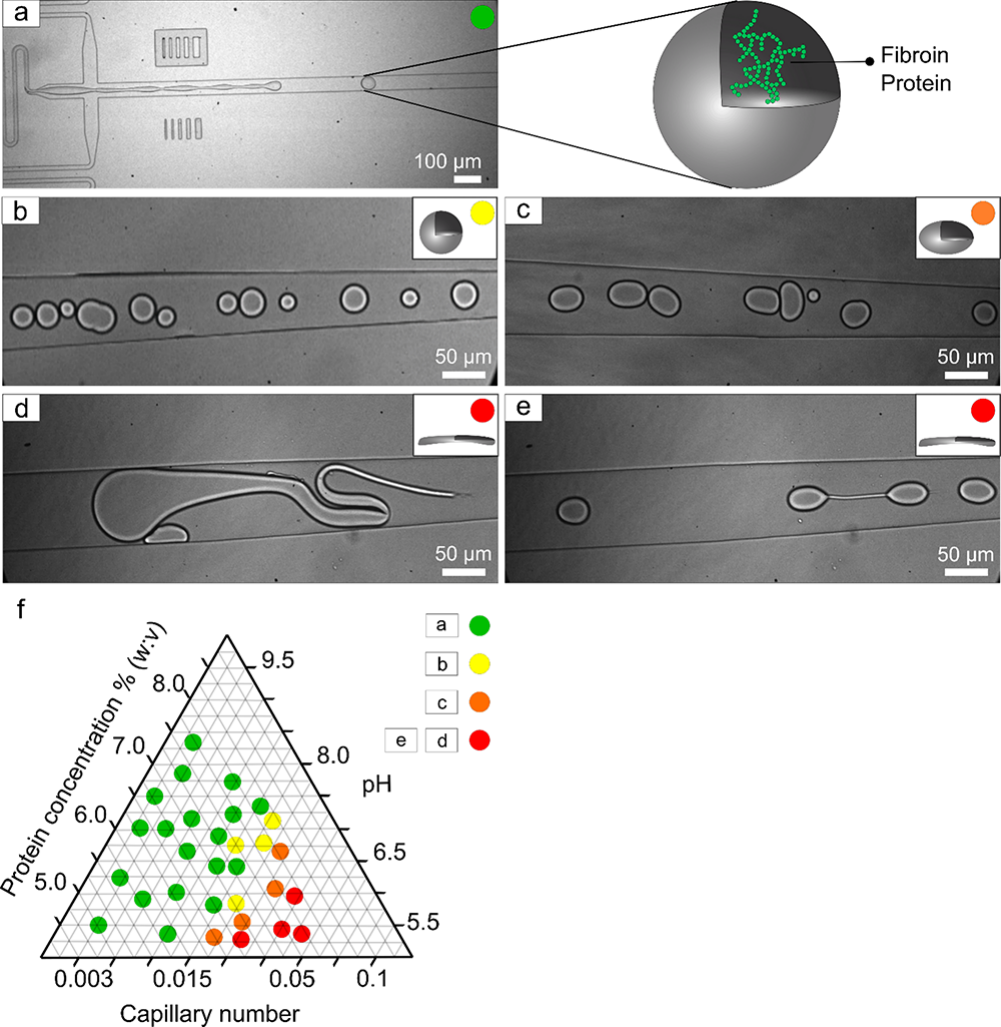
Shape transitions in silk microcompartments. (a) (left) Experimental picture depicting of the generation of the microcompartments in a cross-section microfludic chip. The green label defines a linear mechanical response. (Right) Schematic of homogeneously distributed fibroin inside a spherical microcompartment, where the schematics of the hydrophobic repetitive domain is marked with green and other domains marked with reds. (b) Spherical polydisperse microcompartments demonstrating the conditions triggering the nonmechanical linearity and labeled in yellow. (c) Stable elongated microcompartments labeled in orange. (d and e) Highly inhomogeneous fiber-like microcompartments marked in red. (f) Triangular plot resuming the environmental and fluidic conditions to generate specific microcompartments (sphere, cylinder, and fiber).

We utilized a microfluidic droplet maker approach for generating microcompartments made of reconstituted silk fibroin (see the Experimental Section) under controlled settings. To this end, the aqueous silk fibroin solution (RSF) was flowed through the central channel of the microfluidic droplet maker device and broken into micron-scale droplets (“droplets” here are defined as freshly formed microcompartments inside the microfluidic device) by the continuous oil phase, as depicted in Figure 2a. We focused our initial investigation on conditions favoring the formation of either the spherical or elongated shapes of the droplets as well as conditions enforcing the droplet’s shape transitions (Figure 2b–e). Generally, the transitions between the spherical (Figure 2a,b) and elongated shapes (Figure 2c,d) of the microfluidically formed droplets are balanced by the capillary number (*Ca*) and the flow rates (applied shear).^36^ The *Ca* is highly dependent on the fluid characteristics, such as the viscosity of the starting bulk solution. When the initial fluid that is subjected to encapsulation is defined as a Newtonian fluid, it leads to the formation of droplets with a spherical shape and with dimensionalities (size) inversely proportional to the applied shear,^37^ namely, the greater the applied shear, the smaller the size of the formed droplets. For non-Newtonian fluids, the shape of the droplets might vary from a spherical to a more elongated shape. Imposing the velocity field, the shear force acting on the object is defined by the nondimensional *Ca* number expressed as , where μ, σ, and *V* are the dynamic viscosity, surface tension, and the velocity of the continuous phase, respectively. Considering the dimensionality of the microfluidic droplet makers used in our experimental settings, the following parameters were used for *Ca* estimation: surface tension σ = 3 mN·m, dynamic viscosity μ = 0.04 mPa·s, and the *V* value calculated from the imposed *Ca*. Thus, for a Newtonian fluid, the squeezing mode (*Ca* < 0.015) is governed by the surface tension forces; this results in the formation of droplets with diameter values larger than the microfluidic channel. In contrast, the dripping mode (*Ca* > 0.015) is governed by the shear stress, and thus, the generated droplets appear with a considerably smaller diameter with respect to the microfluidic channel.^38^ Notably, *Ca* = 0.015 does not define the threshold between the two modes. Therefore, in our experiments, we used *Ca* number 0.003 for the squeezing regime, 0.015 as the transition between regimes and 0.048, and 0.1 for the dripping mode of droplet formation, where a 0.1 value is more sensitive to the applied shear, which we observed from our experimental results and which are described in detail in the following sections. The same *Ca* numbers were also used for estimating the velocity fields.

For the microfluidically formed silk droplets, we experimentally observed that at squeezing and transition modes (*Ca* ≤ 0.015), the fibroin solution has a quasi-linear shape transition behavior, even when parameters such as pH, protein concentration, and the applied shear were varied. For the dripping mode (*Ca* > 0.015), the effect of the shear became dominant; thus, a gradual transition between a spherical and a cylindrical, fiber-like shape of the droplets was recorded. The intrinsic characteristic of aqueous silk protein is its high instability at low pH values (≤pH 6). Exposing silk to an acidic pH is expected to trigger changes in the protein fold (a transition from a native fold to an aggregative β-sheet-rich structure), which, in turn, can be manifested in viscosity changes in the silk-rich solution. Thus, we probed how the protein concentration and pH changes affect the shape transitions of the microcompartments. It has been previously reported that the pH of the *B. mori* gland lumen ranges from >∼8.2 in the posterior section of the gland^24^ to <∼6^39^ in the anterior section of the gland. The artificial analogue of native silk fibroin, which is RSF, appears in its fully soluble monomeric form at pH 9.0, whereas when exposed to pH < 5, the silk protein adopts an aggregative β-sheet-rich conformation.^40^ We observed that lowering the pH from 9.5 to 5.5 in RSF starting solution (prior to compartment formation), followed by microfluidic compartmentalization, triggers the microcompartment shape transitions from a sphere to a cylinder and then to a fiber-like shape. Increasing the silk fibroin protein concentration from 5 to 7% (w/v) affected only the size of the formed droplets, as shown in Figure 2d,e. Thus, the overall silk microcompartment shapes and shape transitions are balanced by the changes in the independent environmental parameters, as summarized in the ternary plot of Figure 2f.

### Rheological Characteristics of Bulk vs Microcompartmentalized Silk Fibroin Protein

**Figure 3:**
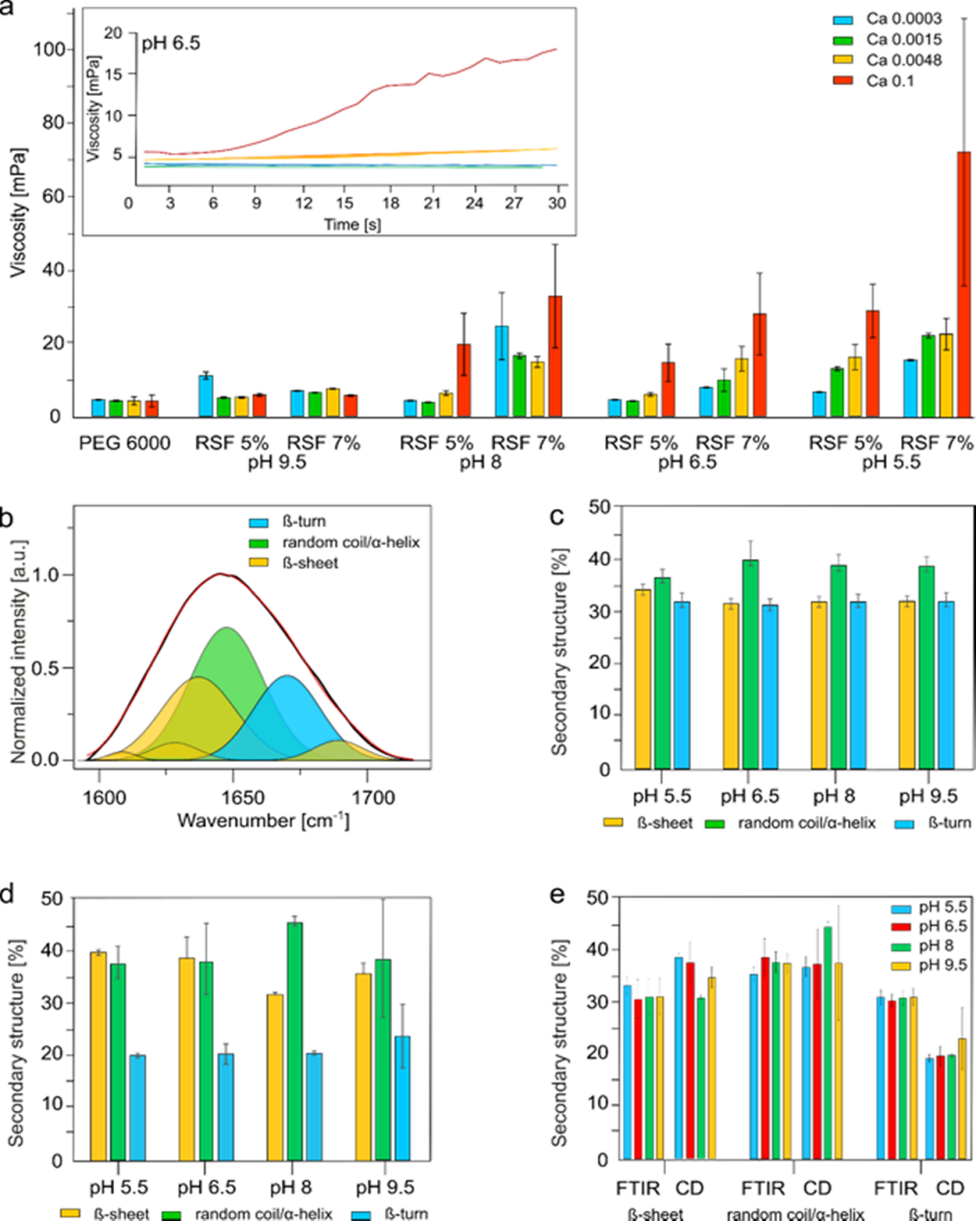
Silk rheology and secondary structure organization. (a) Chart summarizing results of dynamic viscosity measurements using the rheometer under the shear values calculated at the microfluidic chip wall for the different fluid dynamic conditions. The experiments were performed keeping a constant strain rate over time (30 s) and following the changes of dynamic viscosity as reported in the insert (belonging to pH 6.5). Three samples were analyzed for each condition. (b) FTIR analysis addressing the content of β-sheets, α-helix and random coil, and β-turn by means of the seven peaks corresponding to the vibrational wavelengths of the secondary structure. (c) Quantification of the secondary structure organization for the different RSF solutions tested. It is noted that no significant statistical differences between the solutions have been detected. (d) Secondary structure quantification using CD analysis. (e) Structural organization comparison between FTIR and CD analysis.

To further understand the link between the fluid characteristics and the shape transitions in silk microcompartments, we performed a detailed investigation of the viscosity responses of bulk silk protein solution to changes in the applied shear force, concentration, and to changes in pH. To this end, we performed a rheological analysis of bulk aqueous silk solution by applying shear rates equivalent to those acting in a microfluidic droplet maker device during microcompartments formation.^41,42^ Four different capillary numbers were used in the microfluidic chip; each of them uniquely defines the shear rate values. Specifically, for *Ca* numbers of 0.003, 0.015, 0.048, and 0.1, the corresponding shear rates are 400, 1600, 4800, and 9600 s^–1^. Thus, we exposed silk protein solutions (5 and 7% w/v) at variable pH values (see the description below) to the abovementioned constant shear rates for 30 s and tracked the consequent changes in silk viscosity (see the inset of Figure 3a). The results are summarized in Figure 3a, showing the mean and standard deviation, whereas the data are organized from a high pH of 9.5 to a low pH of 5.5. For the calibration test, we used a well-characterized aqueous solution of polyethylene glycol (PEG) 6000 kDa, known to have Newtonian viscosity at a concentration of 100 mg/mL, equivalent to the nominal viscosity of aqueous silk fibroin (for both 5 and 7% concentrations). As expected, from the Newtonian fluid, the measured PEG viscosity is constant and is independent of the applied shear rate. When the concentration of silk fibroin protein was kept constant, and the pH value was set to 9.5, we observed no significant effect of shear on silk solution viscosity (Figure 3a). However, when the pH was lowered to 5.5 at high (7%) as well as at low (5%) protein concentrations, the imposed shear had a tremendous effect on silk solution viscosity.^21^ To this end, we performed a systematic study on the effect of variable shear rates and variable pH (from 5.5 to 9.5) and silk protein concentrations (5 and 7%). Importantly, we observed that with an increase in protein concentration and with the lowering of pH, the aqueous silk becomes more responsive to the applied shear, thus resulting in more pronounced changes in viscosity over a shorter time of exposure (see Figure 3a). Such behavior could stem from the structural instability of silk protein at low pH values as a function of time.^22,43^ According to the literature reports, changes in pH from basic to more acidic trigger structural transitions in silk, under the action of shear, from its native fold, predominantly random-coil conformations, into an aggregative β-sheet-rich structure. To further unveil the structural changes of the silk protein in solution as a function of variable pH (see Experimental Section), we performed Fourier transform infrared spectroscopy (FTIR) analysis.

Generally, FTIR analysis enables one to detect the conformational changes in proteins by following the changes in the vibrational band of Amide I (1600–1700 cm^–1^) that correspond to the CO stretching. In this band, for proteins and peptides, the content of the secondary structures is defined by intermolecular β-sheet (1610–1625 cm^–1^), native β-sheet (1625–1635 cm^–1^), random coil or α-helix (1635–1665 cm^–1^), β-turn (1665–1690 cm^–1^), and antiparallel amyloid β-sheet (1690–1705 cm^–1^), presented in the normalized absorption spectra shown in Figure 3b after subtracting water and deconvoluting the signal using the above peaks (Supplementary Figure S4). The obtained results of FTIR analysis are then summarized in Figure 3c. Interestingly, our FTIR analysis revealed no differences in the secondary structure for aqueous silk fibroin solutions at pH 5.5, 6.5, 8 and 9.5, showing the α-helical and random coil contents of ∼32%, β-sheets ∼38%, and β-turn % ∼30%. The results indicate the relatively low sensitivity of silk protein monomers to changes in pH when in a fresh RSF solution. As an additional proof of the secondary structure composition, circular dichroism (CD) analysis was performed. The raw spectra were acquired in the range 180–260 nm^–1^ (Supplementary Figure S5) and further analyzed by using the online software of Dichroweb^44^ described in the Experimental Section. The quantitative results, which are summarized in Figure 3d, show an α-helical and random coil content of ∼40%, β-sheet ∼40%, and β-turn ∼20%. The results from two analytical techniques were compared and are presented in Figure 3e, and notably, no pronounced differences are shown in the secondary structure content. These results point to the instability of the structured fluid only under external stimuli (such as strain rate or shear forces).

### Rheology of Microcompartmentalized Silk Protein

**Figure 4:**
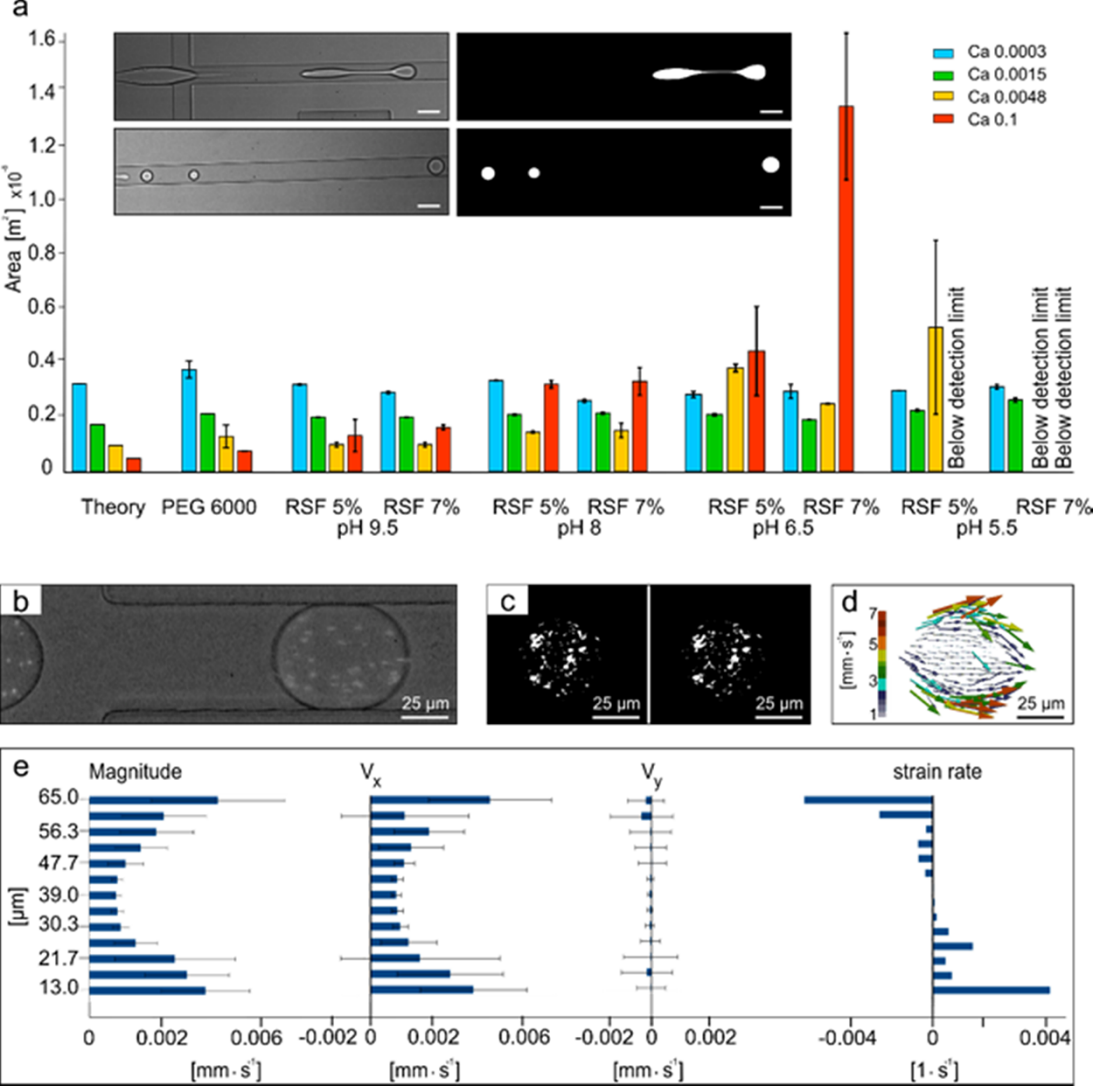
Micro-rheology of silk microcompartments. (a) Area calculation performed by using the home-made ADR algorithm combined with image processing and an object recognition toolbox in matlab. The insets show the results of the whole algorithm for the case of fiber-like (top) and spherical (bottom) microcompartments. At least three microcompartments were analyzed for each conditions (b–d) μ-PIV technique to assess the velocity field inside the RSF compartments: from videos acquired with fast camera (b) to the velocity distribution (d), passing through the auto-correlation algorithm^46^ between two consecutive frames (c). (e) Quantitative assessment (from left to right) of magnitude, *V_x_*, *V_y_*, and the shear strain rate distribution obtained directly from the estimated velocity field.

To standardize the process of silk microcompartmentalization via a microfluidic approach and to be consistent with the analysis, following the work of Chong et al., the droplet dimensionalities were calculated using the automated droplet recognition (ADR) algorithm.^45^ As shown in the inset of Figure 4a, for each video, ADR automatically recognizes the droplets and assesses the geometrical features by quantifying the droplet area (see the Experimental Section for the algorithm’s details and validation) and assuming an axial symmetry of the generated microcompartments. In our study, we demonstrated a correlation between the droplet size, variable environmental parameters, including concentration, pH, and shear and corresponding changes in the compartmentalized fluid behavior. Indeed, the environmental and fluid dynamic conditions strongly affect the geometry and the size of the silk droplets formed inside the microfluidic droplet maker device. The silk fibroin droplet geometry and size were compared with the Newtonian aqueous PEG 6000 kDa solution (Figure 4a) to precisely determine whether the RSF behavior deviates from the linear geometrical behavior of the PEG reference. The PEG solution was concentrated like the previous bulk rheological analysis at 100 mg/mL. Additionally, a numerical justification was conducted by using the lattice Boltzmann method (LBM), as shown in Figure 4a. LBM was introduced as a strategy to confirm the theoretically linear behavior of PEG (see the SI for detailed information). Thus, no pronounced differences with respect to the microfluidically formed microcompartment area of PEG were detected. Furthermore, LBM was used as an additional tool to verify the conditions triggering the formation of complex silk fibroin droplet shapes, which are asymmetrical and bigger than the microcompartments formed from Newtonian fluid (PEG). Specifically, the computational domain was designed by considering the droplet maker microfluidic chip configuration, the fluid characteristics (viscosity and the density ratio between continuous and discontinuous phases) and the fluid dynamic conditions (the *Ca* number). The model provides the theoretical geometrical dimensionalities shown in the Supplementary Movies S3–S6 (for more details on the imposed independent parameters and code validation, see the Experimental Section). Importantly, the full wall hydrophobicity was imposed by simulating the silane surface treatment of the inner part of microfluidic channels.

Microcompartments generated by using the microfluidic approach were labeled and identified as spherical, cylindrical, and fiber-like shapes; they were intrinsically defined as asymmetrical and large compartments. Similar to the results obtained from the bulk analysis of silk fibroin rheology, for both protein concentrations of 5 and 7%, at pH 9.5 and for all *Ca* numbers, there was no pronounced difference in the microfluidically formed droplets’ shape and area. The results indicate that under the combination of the abovementioned environmental parameters (concentrations, pH, and applied shear), a silk fibroin solution behaves as a Newtonian fluid, similar to PEG, which has been confirmed experimentally and supported by LBM simulations. The only detected exception is for *Ca* = 0.1, where generated compartments appear to be more polydisperse in their size (area) and spherical in their shape.

On the other extreme, silk fibroin at pH 5.5 exhibited a deviation from the linear area dependency of PEG droplets. Starting from *Ca* = 0.015, which is defined as a transition mode (from Newtonian to non-Newtonian), we observed the initiation of changes in the shape and area of the RSF droplets. However, for *Ca* > 0.015 (0.048 and 0.1), such variations become more evident. Indeed, for these values, the droplets could not be detected due to the bigger region of interest (ROI). A gradual and less pronounced shape diversity and size complexities were detected for the silk fibroin droplets at pH values of 6.5–8. Interestingly, for compartmentalized silk fibroin at 5% concentration and pH 6.5, the droplets appear predominantly cylindrical in their shape, and fiber-like shapes were observed for 7% protein concentration at pH 6.5. Eventually, for the silk fibroin at pH 8, the spherical and cylindrical shapes were recorded for 5 and 7%, respectively.

We also characterized the encapsulated fluid. To quantitatively analyze the shear acting inside the microfluidic device imposed by the continuous phase, the velocity distribution within the droplets was assessed by means of μ-particle image velocimetry (μ-PIV). The algorithm relies on the experimental performance of suspended fluorescent beads (1 μm in diameter, see the Experimental Section) in the aqueous silk fibroin solution (at a concentration of 0.2%); thus, the velocity distribution inside the droplets can be deciphered. Briefly, μ-PIV is based on an auto-correlation algorithm, and by recursively taking two consecutive frames of a movie (see Figure 4a,b), the algorithm automatically computes the time evolution of the velocity distribution within the droplets (Figure 4c).^46^ Generally, the velocity field (Figure 4d) is considered faithfully followed if and only if the beads have no inertial interference. This is physically true when the Stokes number is less than one as  with *t*_0_, *u*_0_, and *l*_0_ being the relaxation time, fluid velocity, and the characteristic channel dimension. , with ρ, *d*, and μ being the density, the diameter of the fluorescent beads, and the dynamic viscosity of the aqueous silk fibroin solution, respectively.

Unfortunately, the trade-off between the fluorescence signal intensity of the beads and the exposure time leads to acquiring values less than 1000 fps; therefore, the kinematic pathway is precisely followed only at the squeezing mode of the *Ca* number (*Ca* = 0.003). We observed that the strain rate distribution  (calculated in Figure 4d along the vertical axes of symmetry) plays a major role at the interface of the two immiscible fluids, and thus, it locally triggers at first changes in viscosity (Figure 4e), which, in turn, stem from changes in silk protein self-assembly.

### Morphological Analysis of Silk Microcompartments

**Figure 5:**
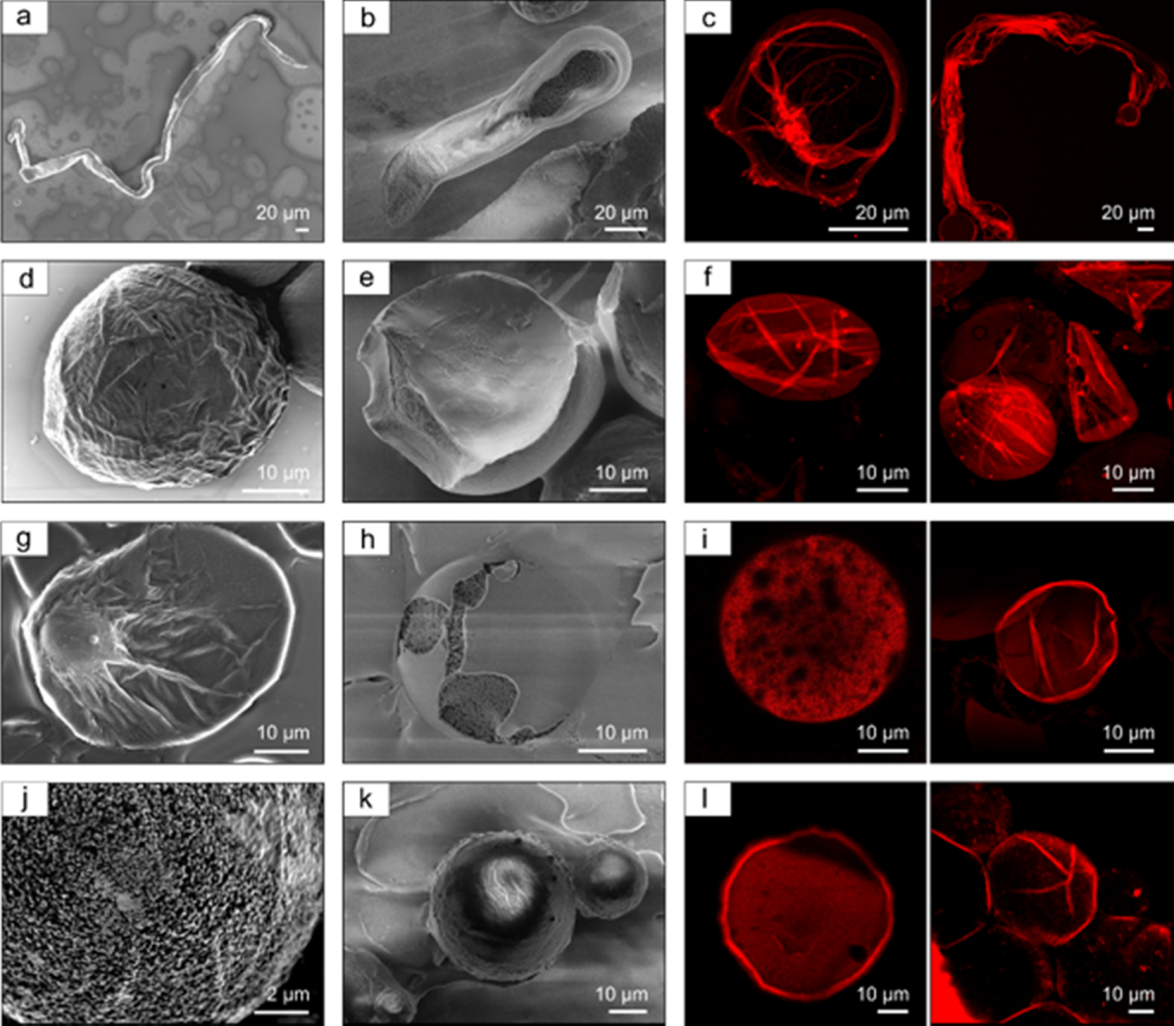
Morphological analysis of silk microcompartments. (a, d, g, j) SEM reveals the wrinkles’ density and distribution on the microcompartment’s surface. The first row shows a magnification of the microcompartment with fiber-like shape formed at pH 5.5. (b, e, h, k) Cryo-SEM shows the surface morphology of the microcompartments. (c, f, i, l) Confocal optical microscopy images present the mesoscopic characterization of the internal microcompartments’ organization visualized by using the fluorescence of Nile Red dye.

Finally, we analyzed the morphology of the microfluidically formed silk microcompartments. The surface morphology of the microcompartments formed under different conditions (variable pH and protein concentrations) and the anisotropic deformation in the form of wrinkles on the compartments’ surface, formed under the acting shear forces,^47,48^ can shed light on the responses of the silk fibroin pulp to the compartmentalization process. To decipher a detailed perspective of the distribution of the outer shell topology of the microfluidically formed silk compartments, scanning electron microscopy (SEM) using an in-lens detector (traditionally used to improve the surficial imaging contrast) was utilized (Figure 5a,d,g,j). Thus, following the protocol of sample preparation (see the Experimental Section), slight shrinkages in the volume of compartments were detected. Interestingly, the shrinkage did not compromise the final shape of the microcompartments. Microcompartments with a fiber-like shape were formed from a silk fibroin solution at pH 5.5, as shown in Figure 5a and Supplementary Figure S6a. Microcompartments with more complex asymmetrical shapes, where, for example, there are two interconnected spheres or cylindrical compartments, were also detected and are depicted in Supplementary Figure S6b. The process of generating these types of asymmetrical compartments is shown in Supplementary Movies S7, S8, and Supplementary Figure S7. Furthermore, in addition to the asymmetrical compartments, we observed the formation of bigger inhomogeneous compartments, as depicted in Supplementary Movie S9. When the pH of the silk stock solution increased from 5.5. to 6.5, the presence of wrinkles on the surface of the microcompartments was observed (whereas the microcompartments’ rheology can be seen in Supplementary Movie S10). Differing from pH 5.5, these microcompartments appear with a cylindrical or ellipsoidal shape. Cryo-SEM revealed the existing variations in the surface morphology and the orientation of the surface deformations (wrinkles) as well as the encapsulated protein pulp distribution. At pH 8, smaller deformations on the surface of microcompartments were observed, which appear even less pronounced at pH 9.5. Cryo-SEM has been used for going deeper inside the geometrical and structural organization of microcompartments (see the cryo-SEM image in Figure 5b,e,h,k). As for the standard SEM, the four microcompartments were formed at different pH and under the same fluid dynamic conditions (*Ca* = 0.1) and concentration (7%). The inhomogeneous shape formations observed at pH 5.5 highlight the existing differences between the outer surface and the internal protein fluid.

A magnification of the structural variation between the interface and the bulk is presented in Supplementary Figures S7 and S8, where the macroscopic surface deformations were also detected; however, the presence of these deformations did not affect the final shape of the formed microcompartments, pointing to the viscoelastic characteristics of the encapsulated fluid. Interestingly, we observed that surface deformations are aligned with the shape axis (for the cylindrical microcompartments, see Figure 5b), pointing to the role of elongational flow acting in a microfluidic channel during the formation of the microcompartment (Supplementary Figure S7), which has also been previously confirmed by μ-PIV analysis. Furthermore, we observed a sharp separation between the outer shell and the core. A similar trend was observed for microcompartments formed at pH 6.5, in which the surface deformations on the outer shell and orientation were detected. At pH 8, the structures present minor surface deformations as in Supplementary Figure S8a. As depicted in Supplementary Figure S8b, on one side, the wrinkles are oriented along the microstructure’s surface and after raising the temperature from −120 to −80° during the cryo-EM scan, the induced etching from the electron beam reveals the internal organization of the microcompartment, which is porous and sponge-like. Eventually, at pH 9.5, the microcompartments exhibit the presence of small surface deformations on the spherical compartment shell.

Next, we used the Nile Red staining assay to reveal whether there are any variations in the density of the encapsulated silk fibroin fluid, similar to the one observed in native silk microcompartments. Indeed, we observed variations in the density of the encapsulated RSF fluid; however, these variations were less pronounced compared with microcompartmentalized native silk. This discrepancy might stem from differences in the protein concentrations. The native silk appears at high concentrations (20%). As previously shown by μ-PIV analysis, given the velocity regime inside microcompartments, the higher shear force acts at the interface, and accordingly, the fibrillation process is also expected to initiate at the interface (Figure 5c,f,i,l and Supplementary Figure S9).

## Conclusions

We described a comprehensive study aimed to reveal the role of the microcompartmentalization phenomenon and the contribution of the dynamically changing chemical environment to the storage-to-spinning transition in a silk fibroin protein solution. We utilized a microfluidic approach for generating artificial analogues of silk microcompartments and probed the impact of changes in each environmental parameter on silk self-assembly. Our results indicate that although the characteristics of the silk protein fluid (Newtonian vs non-Newtonian) are highly dependent on the pH and on the protein concentration, the shape of the compartments is defined by the rheology of silk-rich fluid encapsulated in microcompartments. At a relatively low protein concentration (5%) and a high pH (>9), the protein fold is stabilized and the overall viscous structured silk fluid preserves its Newtonian behavior. Under such conditions, the microcompartments’ geometry is typically spherical and favorable for the silk protein storage, which has been experimentally and theoretically confirmed. The spontaneous silk microcompartmentalization takes place when the protein concentration increases. Thus, gradual lowering of the pH and increasing the silk protein concentration results in a transition from a spherical shape to a more elongated (cylindrical and fiber-like) shape of silk microcompartments. The shape transition is accompanied by initiation of the silk protein self-assembly process and by a switch in the silk fluid characteristics from Newtonian to non-Newtonian. These processes, in nature, are modulated by the elongational flow, similarly to those acting in the anterior-middle and anterior sections of the silkworm silk gland. These events are followed by the microcompartments’ disassembly, solution-to-solid transition of silk protein monomers into silk nanofibrils and further alignment of nanofibrils into micron-scale fibers.

Overall, our results contribute to a better understanding of the silk protein behavior during the storage-to-spinning transition, which is modulated by the combined action of multiple events, including compartmentalization, the dynamically changing chemical environment, and shear forces. We believe that our study opens avenues in understanding the origin of programmable features in silk material.

## Experimental Section

### Fibroin Extraction

*B. mori* cocoons were purchased, peeled, and cut following the protocol^28^ to obtain very thin sheets of cocoon debris for a total of 5 grams. Then, the cocoon pieces are placed in 2 L of boiled water after dissolving 0.02 M of sodium carbonate (Na_2_CO_3_) and boiled two times for 15 min each. After having been cooled down, the cocoon pieces are rinsed with ultra-pure water and left to dry overnight. Lithium bromide (LiBr) (Acros Organics, Thermo Fisher Scientific) at a concentration of 9.3 M for 3 h at 65 °C has been used to solubilize the fibroin protein. Then, the yellowish solution is poured into a 10 kDa dialysis bag (SnakeSkin, Thermofisher) and placed for three days in ultra-pure water at 4 °C. It should be noted that water is changed three times per day. Eventually, the solution is centrifuged (Sorvall LYNX 6000, Thermo Scientific) twice at 12000 rpm, for 20 min at 4 °C and then stored in a refrigerator at 4 °C.

### Fabrication of the Microfluidic Devices

The microfluidic chips are fabricated using standard optical lithography. First, a 2D design is realized using the open-source computer-aided-design (CAD) Layout Editor software. Then, the design is converted into .gdsii format and is ready to be used with the MaskAligner (SussMicrotec-MA6) for photolithography. Briefly, the SU8–2025 (Micro-Chem) negative photoresist is poured onto a clean 4″ silicon wafer and spun at 3000 rpm for 40 s with an acceleration of 300 rpm on a spin coater (Polos Spin 150i) and then soft-baked for 1 min at 65 °C plus 20 min at 95 °C before exposure. After the wafer is baked with 30 μm thickness of photoresist, it is placed on the mask aligner and exposed for 200 mJ/cm^2^. The post-exposure bake is for 5 min at 95 °C. The photoresist is then developed using a PGMEA developer for 8 min and rinsed with IPA for 3 min. Eventually, a salinization treatment is performed using trichloro(1*H*,1*H*,2*H*,2*H*-perfluorooctyl) (Aldrich) to favor detachment during soft lithography. Polymethil-disiloxane (PDMS) is used as a biocompatible material for soft lithography. Briefly, the base curing agent ratio is equal to 10 (w:w), mixed, poured on the silicon master form, left to degas in a vacuum chamber until no bubbles are detected, and then placed in an oven overnight at 65 °C. Once cured, the PDMS is peeled off, and inlets and outlets are punched using a 0.75 mm disposable biopsy puncher (Robbin Instrument) for tube insertion. Then, the ultrasound bath is cleaned by immersing the microfluidic chip in ethanol (70%) for 15 min and placing it in an oven for 15 min and allowing for evaporation. Once the microfluidic chip is fully dried, oxygen plasma bonding is used to permanently attach PDMS with glass slides for 20 s, 4 mW, at 2×10^–3^ Pa. Eventually, to impose full wall hydrophobicity within the final microfluidic chip channels, salinization treatment (using silane as before) is conducted overnight in a vacuum.^49^ The final microfluidic chip is stored at room temperature and sealed with tape to preserve hydrophobicity.

### Compartments’ Rheology: Optical Acquisition

The microfluidic chip is placed on a microscope stage (Axio Observer 7, Zeiss Fluorescent microscope) and connected from the inlets with polythene tubes (Smiths Medical, Thermo Fisher Scientific) to the syringe (Norm-Ject, Thermo Fisher Scientific) through needles (containing the two immiscible solutions: on one side, oil FC-40 (Fluorinert FC-40, Chem Cruz) and surfactant (Ran Biotechnologies) at 2% (w:w) and on the other side, RSF solution. Then, the syringes are connected to the syringe pump (neMESYS Low Pressure Syringe Pump) for precise control of the flow rate. An ultra-fast camera (Ultra High-Speed Camera, Phantom v1212) is used to capture the rheology of the structure generation in the cross-section. Specifically, the acquisition is strictly related to the fluid dynamic conditions as follows:

- 2000 frame per second (fps) for *Ca* = 0.003
- 4000 fps for *Ca* = 0.015
- 10,000 fps for *Ca* = 0.048
- 10,000 fps for *Ca* = 0.1

During the experiments, the structures are collected by connecting the silanic tube from the microfluidic outlet port to an Eppendorf tube. Then, the structures are stored at 4 °C to inhibit additional fibrillation and are ready to be analyzed.

### Optical Microscopy

Fluorescent microscopy is used to analyze structures generated using microfluidics and using a rheometer. The samples are prepared using home-made PDMS wells firmly attached to a glass slide (Bar Naor, plain slides 26 × 76 × 1 mm). Briefly, the PDMS having a ratio base–curing agent equal to 10 (w:w) is spun (Polos200 Advanced) at 300 rpm for 60 s and then left in the oven at 65 °C for 3 h. Once curing takes place, the wells are obtained using a 3 mm puncher. Then, structures are placed in the PDMS wells and stained with Nile Red dye (Acros Organics, Holland Moran) at a concentration of 0.1% (v:v) and sealed with another glass slide attached on top to inhibit the evaporation. The acquisitions are obtained using confocal microscopy (Zeiss LSM 800 with Airyscan) and specifically, from Nile Red staining, polar and nonpolar regions are excited with a laser wavelength of 564 nm (green) and 515 nm (blue), respectively. Intrinsic fluorescence is detected using an excitation wavelength of 385 nm (near-UV).

### Scanning Electron Microscopy (SEM) Analysis

After collection in a microfluidic chip, the RSF structures are still immersed in oil and surfactant; therefore, the compartments are rinsed with pure FC-40 to remove any surfactant residue and then rinsed with ultrapure water. Then, 1 μL of compartment solution is poured onto a clean 0.5 × 0.5 cm^2^ silicon wafer and immersed in liquid nitrogen for a few seconds and then lyophilized overnight. A chrome deposition of 5 nm is performed with sputter before SEM acquisition to avoid any induced damage to the microstructures.

### Cryo-Scanning Electron Microscopy (Cryo-SEM) Analysis

Cryo-SEM imaging was performed in two steps: high-pressure freezing and freeze-fracture.

The first step requires sample preparation. Microstructures immersed in oil were rinsed with pure water, and an aliquot of 2 μL was sandwiched between two metal discs 3 mm in diameter and with two different cavity heights of 50 and 100 μm. It should be noted that the inner disc surface was scratched with a razor blade to favor the adhesion between misstructures and the surface. Moreover, two different disc cavity heights were used according to the variability in the microstructure’s dimensionalities. Then, 2 μL of PEG solution was added to the disc cavity, facilitating the attachment and confinement of microstructures to the bottom surface. Immediately after matching the two discs, cryoimmobilization was performed using a high-pressure freezing device (HPM10; Bal-Tec AG). Frozen conditions were maintained by storing the disc in liquid nitrogen.

Freeze-fracture (BAF 60; Leica Microsystems) was conducted by transferring the sample with the vacuum cryotransfer (VCT 100; Leica Microsystems). The fracture was performed at a temperature of −120 °C; then, the sample was shifted and placed in the Ultra 55 SEM (Zeiss) chamber under the same temperature conditions. Samples were observed at −120 °C. In some cases, such as those presenting high microstructure complexity or only a partial fracture, sublimation (etching) was required by reducing the temperature to a value of −80 °C. A secondary electron in-lens detector was used to image the microstructures at a voltage of 2–4 keV.

### Analysis of the Silk Protein Secondary Structure

FTIR spectroscopy was used to define the RSF solution secondary structure distribution in the amide band I. Samples were analyzed using a Nicolet iS50 FT-IR spectrometer by mounting an ATR Smart iTX. The fibroin solution was assessed in its liquid form. At least three different samples were analyzed for each solution with different pH values. The absorption infrared spectrum (from 400 to 4000 cm^–1^) was assessed with a resolution of 4 cm^–1^ and then underwent 32 independent scans for each measurement. The spectra of the solvent and air backgrounds were independently collected and then subtracted from the RSF samples before further post-processing. Post-processing was performed using OriginPro software. First, raw data were extrapolated in amide band I (1595–1720 cm^–1^), subtracting baseline and normalizing the profile to 1. In this range, the second derivative was calculated and deconvoluted by selecting the seven Gaussian peaks at the following wavelengths corresponding to the vibrational wavenumbers: 1609, 1621, 1631, 1650, 1695, and 1703 cm^–1^.^50−52^ The seven Gaussians approximate the absorption spectra for convergences having a chi-square tolerance value <1 × 10^–6^. After deconvolution, the secondary structures are classified as follows: 1609, 1621, and 1631 cm^–1^ for intermolecular β-sheets; 1650 cm^–1^ for α-helix and random coil, 1673 cm^–1^ for β-turn; and 1695 and 1703 cm^–1^ for antiparallel amyloid β-sheets.^52^

Circular dichroism (CD) spectra were collected in the 185–250 nm range using a J-715 spectropolarimeter (Jasco, Tokyo, Japan) having a data resolution of 0.5 nm. The RSF solutions were diluted until a concentration of 0.01 mg/mL was reached, and a 0.1 mm quartz cuvette was used. The secondary structure content was analyzed using the free online software DichoWeb.^44^ Continl was the algorithm used to quantitatively assess the structural content. The results were obtained as a percentage of β-sheets, β-turn, α-helix, and unstructured content.

### Rheological Analysis

A rheometer (Discovery HR-2, TA Instruments) is used with a cone geometry (0.5° inclination) and is mounted for measurements. The cone geometry was chosen to keep uniform shear forces across the sample.

Before the experiment, an RSF aliquot of 500 μL is taken from the stock solution and placed in the vacuum chamber for 30 min to degas. After degassing, 200 μL of solution is placed on the rheometer stage and the cone geometry is approached until a gap of 17 μm is reached. Then, the sample is left to relax for ∼15 min before running the experiment.

Viscosity is measured following the same shear rate values generated in the microfluidic cross-section.

The measurement consists of a series of alternate steps, quantitative calculation of viscosity measurements, and qualitative solution behavior (i.e., viscoelastic or gel) with a frequency sweep as follows:

1. Amplitude sweep [0.002–10] strain at 1 Hz
2. Frequency sweep [0.02–100] rad/s at 0.01strain of amplitude
3. Viscosity measurements for 60 s at 1 1/s
4. Viscosity measurements for 60 s at 400 1/s
5. Frequency sweep [0.02–100] rad/s at 0.01 strain of amplitude
6. Viscosity measurements for 60 s at 1600 1/s
7. Frequency sweep [0.02–100] rad/s at 0.01 strain of amplitude
8. Viscosity measurements for 60 s at 4800 1/s
9. Frequency sweep [0.02–100] rad/s at 0.01 strain of amplitude
10. Viscosity measurements for 60 s at 9600 1/s
11. Frequency sweep [0.02–100] rad/s at 0.01 strain of amplitude

Note that between each step, 30 s of conditioning are imposed.

After the experiment, the samples are collected using pipettes and immediately analyzed (i.e., microscopy, SEM).

## Supporting Information Available

Additional experimental methods; flow chart of the automatic estimation of microcompartment dimensionalities (i.e., automated droplet recognition) algorithm with “Case of Validation”; Lattice Boltzmann Method with the Ginzburg–Landau free energy functional theoretical model including the “Case of Validation”; and MATLAB image post-processing (PDF)
Silk gland cut at its posterior-middle section for further analysis (MP4)
Silk gland cut at posterior-middle section for optical microscopy analysis (MP4)
Fluid dynamic simulations using Lattice Boltzmann Method with Ginzburg–Landau free energy functional for Newtonian fluids (AVI)
Computational domain designed by considering the droplet maker microfluidic chip configuration, the fluid characteristics (viscosity and the density ratio between continuous and discontinuous phases), and the fluid dynamic conditions (the *Ca* number) (AVI)
Computational domain designed by considering the droplet maker microfluidic chip configuration, the fluid characteristics (viscosity and the density ratio between continuous and discontinuous phases), and the fluid dynamic conditions (the *Ca* number) (AVI)
Computational domain designed by considering the droplet maker microfluidic chip configuration, the fluid characteristics (viscosity and the density ratio between continuous and discontinuous phases), and the fluid dynamic conditions (the *Ca* number) (AVI)
Rheology of asymmetrical silk compartments at pH > 5.5 (MP4)
Process of generating the types of asymmetrical compartments (MP4)
Inhomogeneous asymmetric silk compartments at pH 5.5 (AVI)
Rheology of silk microcompartments at pH 6.5 (AVI)

## Supplementary Material

la3c00354_si_003.mp4

la3c00354_si_004.avi

la3c00354_si_005.avi

la3c00354_si_006.avi

la3c00354_si_007.avi

la3c00354_si_009.mp4

la3c00354_si_010.avi

la3c00354_si_011.avi
