# Supplementary material for: Microcompartmentalization Controls Silk Feedstock Rheology
Source: Langmuir. 2023 Jun 21;39(26):8984–95. doi: 10.1021/acs.langmuir.3c00354 (PMC10324394; doi:10.1021/acs.langmuir.3c00354)
Supplement: Supplementary file 1 — la3c00354_si_001.pdf [file la3c00354_si_001.pdf]

Supplementary Information.

Microcompartmentalisation controls silk feedstock rheology

Marco Elvino Miali[1], Dror Eliaz[1,] Eliaz[1], Aleksei Solomonov[1], and Ulyana Shimanovich[1]*

[1]Department of Molecular Chemistry and Materials Science, Weizmann Institute of Science, 7610001 Rehovot, Israel

** To whom correspondence should be addressed:* ulyana.shimanovich@weizmann.ac.il

Automated Droplet Measurements (ADM)

Following the work of Chong et al.,[1] the automated droplet measurement algorithm requires a series of steps following the order of the background Extraction Operation (BEO), preliminary background removal (BPR) and binary threshold value selection (BTVS).

Briefly, BEO relies on a modified statistical method. The procedure is described below and in **supplementary Figure S10**:

1. An average image (A_{ave}) derived from a sufficiently high number of frames (N_{ave} >40) is generated.
2. An $h = h(F_{ij,n}, R_{n-1}, A_{ij,n})$ algorithm is then applied to each consecutive frame in the range [N_{ave} - $2 \times N_{ave}$] as follows, called preliminary background removal (PBR):
   a. $D_{ij,n}(F_{ij,n}, A_{ij,n}) = \begin{cases} 255, & (F_{ij,n}) > (A_{ij,n}) \\ 255 - (A_{ij.n} - F_{ij,n}), & \text{otherwise} \end{cases}$
   b. After the binarization of $D_{i,j}$ in B_1 and B_2 (one is the complement of the other), the generation of two matrixes, $M_1$and M_2, is performed as:
      i. $M1_{ij,n} = B1_{ij,n} \times F_{ij,n}$
      ii. $M2_{ij,n} = B2_{ij,n} \times R_{ij,n-1}$
   c. $Rij_{,n} = M1_{ij,n} + M2_{ij,n},$

where $A_{ij,n}$, $D_{ij,n}$ and $F_{ij,n}$ are the average matrix, the modifier matrix for PBR and the nth frame inside the recursive loop $[N_{ave} - \ 2 \times N_{ave}]$. Note that i,j represent the matrix components.

The BTVS algorithm determines the best binarization threshold for object recognition. Therefore, for each threshold value in the range of 0-255 the geometries are quantitatively calculated and subsequently assessed (e.g., the area, circularity and perimeter), leading to a sub-range of valid solutions. Then, within the sub-range the selected threshold value is defined by calculating the average.

The Case of Validation

The ADR algorithm was tested using Newtonian fluid polymer PEG 6000 KDa with a concentration of 100 $[\frac{mg}{ml}]$ in pure water. As previously explained, the algorithm detects the compartment's dimensionalities over time. The geometrical assessment is then coupled to the compartment's dynamics. Indeed, using image processing toolbox functions (Matlab Script), additional information regarding the centre of mass velocity, the minor and major axis length and the circularity are easily calculated. These additional parameters have been used to verify the automatic measurement reliability. In **supplementary Figure S11**, the mechanical dynamic viscosity measurements were performed, using a rheometer for four different cases: PEG 6000 KDa having a concentration of 100 $[\frac{mg}{ml}]$ and 400 $[\frac{mg}{ml}]$ and PEG 35000 KDa having a concentration of 100 $[\frac{mg}{ml}]$ and 400 $[\frac{mg}{ml}]$. The dynamic viscosity of a PEG solution was calculated for the three different shear rates belonging to the squeezing (Ca<0.015), transition (Ca~0.015) and dripping (Ca>0.015) modes. The results indicated the same value of dynamic viscosity for all shear rates, confirming the linear nature of the material and the reliability of such measurements.

The ADR algorithm exhibited a high accuracy in detecting the physical area. Following the work of,[2] the compartment's dimensions are inversely proportional to the dynamic solution's viscosity. Therefore, a decreasing trend of the compartment's dimensionality was found. According to this assumption, for the squeezing and transition mode, the droplet minor axis is comparable to the microfluidic chip characteristic length scale. Regarding the

dripping mode, the compartment's minor axis (equivalent to the major axis) is smaller than the characteristic channel length (70 μm), as shown in the bottom right graph. An area estimation follows the same trend as the minor axis length. Fluid dynamic consideration can be performed knowing the frame per second (fps) acquisition and calculating, for each frame, the compartment's centre of mass coordinates. Theoretical velocities are compared to the experimental results. The theoretical velocity does not consider the mass of the compartment; thus, the experimental results showed lower values.

Fluid dynamic modelling

Ginzburg-Landau functional free energy

The multiphase system is modelled on the basis of the Ginzburg-Landau free energy[3] functional as follows:

$$F(\varphi,\Delta\varphi) = \int \psi(\varphi) + \frac{1}{2}k|\Delta\varphi|^2 + \rho c_s^2 ln\rho \, dV \qquad \text{(S1)}$$

Here, $\varphi = \rho_A - \rho_B$ is the ordered parameter describing the normalised density difference between the two fluids and assumes a value equal to ± 1 in the bulk, $\rho = \rho_A + \rho_B$ is the total density and $\psi(\varphi)$ is the bulk free-energy having the form $\psi(\varphi) = \frac{1}{4}a(\varphi^2 - 1)^2$; the term $\frac{1}{2}k|\Delta\varphi|^2$ is the interfacial energy density; it is indirectly related to the interfacial tension, and the $\rho c_s^2 ln\rho$ component emphasises the incompressibility. The chemical potential is assessed as the free energy functional derivative with respect to φ, namely:

$$\mu = \frac{\delta F}{\delta\varphi} = a\varphi^3 - a\varphi - k\nabla^2\varphi \qquad \text{(S2)}$$

Note that a and k are negative and positive parameters, respectively, and that μ assumes a value equal to zero once the interface is at the equilibrium point.

The dimensionless interfacial tension is given by:

$$\sigma = \frac{4k\varphi_0}{3\xi} \qquad \text{(S3)}$$

whereas the interface thickness was defined as:

$$\xi = \sqrt{\frac{2k}{-a}} \qquad \text{(S4)}$$

Importantly, the interface thickness and tension are modelled *via* the parameters k and a.

Lattice Boltzmann Method (LBM)

The temporal evolution of the ordered parameters using the free energy approach is characterised using two distribution functions, namely, f_i and g_i.

$$f_i(\boldsymbol{x} + \boldsymbol{e}_i\Delta t, t + \Delta t) - f_i(\boldsymbol{x}, t) = -\frac{\Delta t}{\tau(\varphi)}\left[f_i(\boldsymbol{x}, t) - f_i^{eq}(\boldsymbol{x}, t)\right] + F_i \qquad (S5)$$

$$g_i(\boldsymbol{x} + \boldsymbol{e}_i\Delta t, t + \Delta t) - g_i(\boldsymbol{x}, t) = -\frac{\Delta t}{\tau}\left[g_i(\boldsymbol{x}, t) - g_i^{eq}(\boldsymbol{x}, t)\right] \qquad (S6)$$

Following the work of Y. Shi et al.,[4] the interfacial forcing distribution is set as

$$F_i = \left(1 - \frac{1}{2\tau(\varphi)}\right) \times \left(\frac{(e_i - u)}{c_s^2} + \frac{(e_i \cdot u)}{c_s^4}\right) \cdot \boldsymbol{f}(\mu, \nabla\varphi)\Delta t \qquad (S7)$$

Having $\tau(\varphi)$ and τ as two independent relaxation time parameters and $\boldsymbol{f}(\mu, \nabla\varphi) = \mu\nabla\varphi$ as the forcing term is directly related to the free energy and the ordered parameter gradient. Note that $\tau(\varphi)$ is directly calculated from $\nu(\varphi)$ as:

$$\tau(\varphi) = \frac{\nu(\varphi)}{c_s^2\,\Delta t} + \frac{1}{2} \qquad (S8)$$

and $\nu(\varphi)$, defined as a linear function of the ordered parameter, considers the viscosity differences between the two fluids as follows:

$$\nu(\varphi) = \frac{1 - \varphi}{2}\nu_a + \frac{1 + \varphi}{2}\nu_a \qquad (S9)$$

The two local equilibrium distribution functions are defined as follows:

$$f_i^{eq}(\boldsymbol{x}, t) = \omega_i\left[A_i + \rho\left(\frac{1}{c_s^2}(\boldsymbol{e}_i \cdot \boldsymbol{u}) + \frac{1}{2c_s^4}(\boldsymbol{e}_i \cdot \boldsymbol{u})^{\mathbf{2}} - \frac{1}{2c_s^2}\boldsymbol{u}^2\right)\right] \qquad (S10)$$

$$g_i^{eq}(\boldsymbol{x}, t) = \omega_i\left[B_i + \varphi\left(\frac{1}{c_s^2}(\boldsymbol{e}_i \cdot \boldsymbol{u}) + \frac{1}{2c_s^4}(\boldsymbol{e}_i \cdot \boldsymbol{u})^{\mathbf{2}} - \frac{1}{2c_s^2}\boldsymbol{u}^2\right)\right] \qquad (S11)$$

with the coefficients A_i and B_i calculated as:

$$A_0 = \left(\rho - \frac{(1 - \omega_0)p}{c_s^2}\right)\frac{1}{\omega_0}; \qquad A_{1-8} = \frac{p}{c_s^2}; \qquad (S12)$$

$$B_0 = \left(\varphi - \frac{(1-\omega_0)\Gamma\mu}{c_s^2}\right)\frac{1}{\omega_0}; \qquad B_{1-8} = \frac{\Gamma\mu}{c_s^2}\ , \qquad (S13)$$

where $\Gamma = \frac{M}{(\tau - 0.5)\Delta t}$ is a coefficient related to the mobility $M = \frac{u_c W_c}{-aPe}$ with u_c the inlet velocity, W_c the inlet width and Pe the Peclet number.

The 2DQ9 lattice vectors are also defined as:

$$\boldsymbol{e_i} = \begin{cases} (0,0), & if\ i = 0 \\ \left(\cos\left(\frac{(\mathrm{i}-1)\pi}{2}\right), \sin\frac{(i-1)\pi}{2}\right) & if = 1-4 \\ \sqrt{2}\left(\cos\left(\frac{(2\mathrm{i}-9)\pi}{4}\right), \sin\frac{(2i-9)\pi}{4}\right) & if = 5-8 \end{cases} \qquad (S14)$$

with $\omega_0 = 4/9$, $\omega_{1-6} = 1/9$ and $\omega_{7-18} = 1/36$.

The macroscopic variables ρ, $\rho\boldsymbol{u}$ and φ are recovered as

$$\rho = \sum_i f_i \qquad (S15)$$

$$\rho = \sum_i f_i \boldsymbol{e}_i + \frac{\mu\nabla\varphi\Delta t}{2} \qquad (S16)$$

$$\varphi = \sum_i g_i \qquad (S17)$$

whereas the Navier-Stokes and the Cahn-Hilliard equations are obtained, with a second order accuracy, using the Chapman-Enskog expansion,

$$\nabla \cdot \boldsymbol{u} = 0 \qquad (S18)$$

$$\rho(\partial_t \boldsymbol{u} + \boldsymbol{u} \cdot \nabla\boldsymbol{u}) = -\nabla p + \nabla \cdot \left(\nu(\nabla\boldsymbol{u} + \nabla\boldsymbol{u}^T)\right) + \mu\nabla\varphi \qquad (S19)$$

$$\partial_t\varphi + \boldsymbol{u} \cdot \nabla\varphi = \nabla \cdot (M\nabla\mu) \qquad (S20)$$

Boundary Conditions (BC)

No-slip boundary conditions are imposed at the wall. To prevent mass leakage, an effect particularly evident at a low Reynolds number, the half-way bounce back is used. Briefly, the fluid domain is divided into fluid nodes and wall nodes; therefore, the boundary conditions are imposed as follows:

$$f_{i'}(\boldsymbol{x}, t + \Delta t) = \begin{cases} f_i^+(\boldsymbol{x}, t) + 2\,\omega_i \rho(\boldsymbol{x}, \boldsymbol{t})\frac{\boldsymbol{e}_i \cdot \boldsymbol{u}_{wall}}{c_s^2}, & \text{boundary node} \end{cases} \qquad (S21)$$

$$g_{i'}(\boldsymbol{x},t+\Delta t) = \begin{cases} g_i^+(\boldsymbol{x},t) + 2\,\omega_i \rho(\boldsymbol{x},\boldsymbol{t}) \dfrac{\boldsymbol{e}_i \cdot \boldsymbol{u}_{wall}}{c_s^2}, & \text{boundary nodes} \end{cases} \qquad (S22)$$

where $\boldsymbol{e}_{i'} = -\boldsymbol{e}_i$ and f_i^+ is the post-collision distribution function.

Wetting characteristics were calculated by considering the wall as a mixture of the two fluids; therefore, only the value of the ordered parameter, φ_w,was imposed. This leads to identifying the chemical potential between the fluid nodes and the neighbouring wall nodes, generating active forces between the fluid and wall. Therefore, the wettability can be addressed using Young's equation, which associates the interfacial tension with the contact angle. Moreover, for static conditions, a droplet attached to the wall defines a contact angle between two fluids *a* and *b* as

$$\cos(\theta) = \frac{\sigma_{b,w} - \sigma_{a,w}}{\sigma_{a,b}} \qquad (S23)$$

Defining the interfacial tension as a function of the ordered parameter and with $\varphi_{wall} = \varphi_0 \tanh\left(\frac{x_{wall}}{\xi}\right)$, the contact angle calculation becomes

$$\cos(\theta) = \frac{3\varphi_{wall}}{2\ \varphi_0}\left(1 - \frac{1}{3}\left(\frac{\varphi_{wall}}{\varphi_0}\right)^2\right) \qquad (S24)$$

Validation of the rheological analysis

The following simulations predict the behaviour of linear PEG 6000 KDa at a concentration of 100 $[\frac{mg}{ml}]$. The choice of this material at this concentration is mainly due to the similar mechanical properties of the RSF solution (5% [w:v]) at a low shear rate, as from the rheological measurement shown in **Figure 3a.** The model was validated by calculating the contact angle on a wall having a different degree of hydrophobicity and considering the propensity of spherical droplet deformation in a Tailor deformation cavity. Additionally, the droplets formed on the microfluidic chip were modelled by imposing the boundary (full hydrophobicity) and the initial conditions (inlet flow rates), which were experimentally demonstrated.

Contact angle measurements

A lattice grid of 100x100 was used as a computational domain to quantitatively assess the contact angle of a semi-circular droplet having a radius equal to 20 cells adhering to a flat wall. The dynamic viscosity ratio is $\frac{\eta_d}{\eta_c} = 1$, $\rho_c = \rho_c = 1000\,\frac{Kg}{m^3}$ and $\eta_c = \eta_c = 10^{-3}\,Pa$. A zero velocity regime was set as in the initial conditions. Periodic boundary conditions (no wall) are imposed in the x-direction, whereas along the y-direction (bottom and top), it bounces back half-way when the specific φ_w value is set. The post-processing is performed after the solution convergency is achieved, as presented in **supplementary Figure S12**. Note that the post-processing is analysed using matlab and that it automatically calculates the contact angle between the compartment at the wall. Interestingly, the compartment detaches completely from the wall only if total hydrophobicity ($\varphi_w = 1$) is assumed.

From eq. 24, the linear contact angle is modulated via φ_w and in **supplementary Figure S12** the φ distribution is shown across the computational domain and presents the droplet profile on the wall. The theoretical contact angle (a continuous line) was compared with the computational results (blue dots) in the top right **supplementary Figure S12.**

"Taylor" deformation under shear-induced flow

Droplet deformation is calculated using the *Taylor* deformation in a binary model. More specifically, a droplet having a diameter of 20 cells is placed into a computational grid of 80 x 80. The deformation is calculated by applying a uniform shear field by moving the parallel plates (top and bottom) in opposite directions (**supplementary Figure S13**) and following the relation $\gamma = \frac{u_w}{h}$ with γ the shear stress, u_w the wall velocity and *h* the distance between the walls. However, the viscosity ratio $\frac{\eta_d}{\eta_c} = 1$ and the densities are $\rho_c = \rho_c = 1$. The periodic boundary conditions are imposed along the x-direction and are bounced back half-way along the y-direction. Eventually, the deformation is expressed by correlating the non-dimensional Capillary number, $= \frac{\gamma R \eta}{\sigma}$, with *R,* the droplet radius, η, the dynamic viscosity and σ, the interfacial tension, with $\Delta = \frac{L - B}{L + B}$, where *L* and *B* are the major and the minor lengths of the deformed droplet. As depicted in **supplementary Figure S13** at a low capillary number, the deformation is linear, and it turns non-linear for Ca>1. At Ca=3 the

droplet breaks up into two smaller droplets, as seen in **supplementary Figure S13** (bottom right) after ~100,000 iterations.

Case of Validation: Predicting the droplet dimensionalities using LBM

The LBM home-made code was tested to simulate the droplet dimensionalities for Newtonian linear fluid. The computational densities and the viscosities were imposed as in the previous paragraph. After creating the computational domain, the initial fluid dynamic conditions were imposed, such as the parabolic profiles for the continuous and discontinuous phase, keeping the same experimental ratio of 1:8 (discontinuous:continuous phase). The experimental campaign showed the droplet dimensionality differences for the squeezing (*Ca*<0.015), transition (*Ca*~0.015) and the dripping (*Ca*>0.015) modes. Therefore, to convert the physical material's mechanical properties into the equivalent adimensional parameters, four different simulations were performed, replicating the same fluid dynamic conditions as those used to study the non-linear behaviour of the fibroin solution. As demonstrated in **supplementary Figure S14a**, the velocity parameters, V_x and V_y, precisely describe the parabolic velocity profile typical of a microfluidic channel. The pictures presented in **supplementary Figure S14b** show the strong analogy between the experimental data and the simulation results. From the experiments, the area quantification uses the same ADR algorithm analysis, whereas for the modelling, only those values of $\varphi < 0$ were used. Notably, the simulated droplet always underestimated the experimental value. This is because the continuity of the ordered parameter function provides a finite thickness at the interface (proportional to the *Peclet* number) between the two immiscible fluids. Therefore, as recognized in s**upplementary Figure 14c**, the absolute numbers are lower, whereas the decreasing trend faithfully follows the experimental data. The table in **supplementary Figure S14c** shows the fluid dynamic non-dimensional *Ca* and *Re* numbers.

Object detection and geometrical assessment

The experimental and numerical results were quantified and investigated using the same "object recognition" algorithm found in matlab. The movies were exported in *.avi* format and directly loaded in matlab, and then using the image processing toolbox functions, the following algorithm was applied:

- *bwlabel* to identify the number of objects for each region
- *bwboundaries* to identify the perimeters of each object and to generate a binary 2D image as input for the next function
- *Regionprop* to define all the geometrical features of each object such as a*rea, major axis length, minor axis length, circularity, centre and perimeter*

Note that all the non-dimensional parameters are transformed into dimensional parameters by calculating the *mmpix* size. Eventually, the compartment's velocity is estimated knowing the centre displacement of each object and the fps.

Supplementary Figures

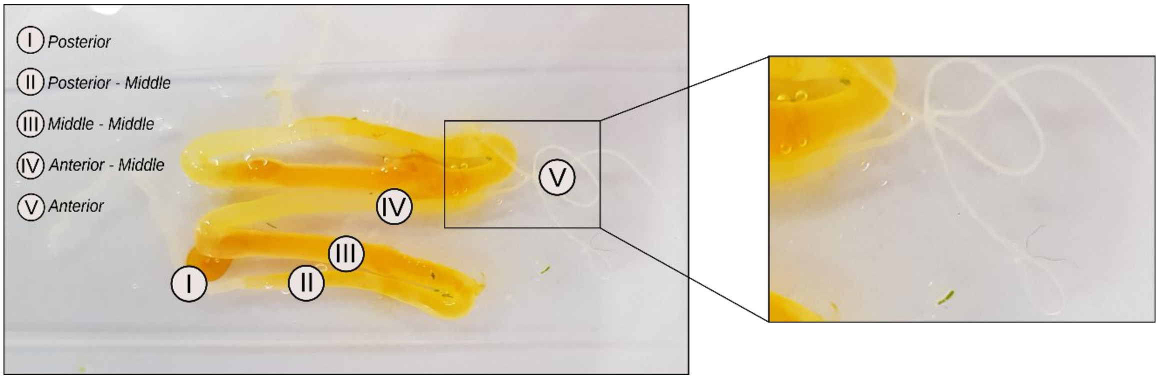

Supplementary Figure S1. *Bombyx Mori silk gland.* *The roman numbers represent the different regions of the silk gland order following the natural silk fluid flow. On the right is a magnification of the anterior region in which the silk undergoes an elongational flow.*

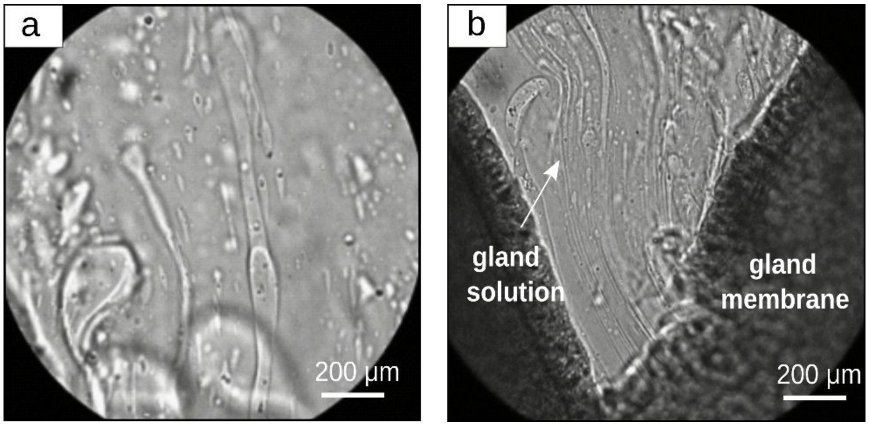

Supplementary Figure S2. ***Bombyx Mori silk gland after performing a cut orthogonal to the silk gland flow***. ***a)*** *Picture in the cut proximity with low shear showing the silkworm multiphase fluid rich-in microcompartments.* ***b)*** *Picture showing the cut section experiencing a higher shear rate, manifested by the more elongated density phases rich-in microcompartments.*

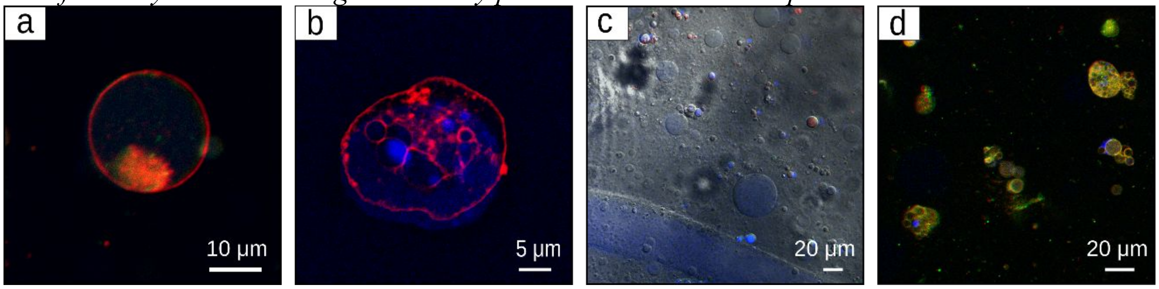

Supplementary Figure S3. *Confocal optical microscopy of native silk gland microcompartments. The pictures were acquired after cutting the silk gland (**Supplementary Figure S2**) and staining with Nile Red. The rich-in microcompartments exhibit a high inhomogeneity of the silk protein distribution (**a,b**) and show pronounced variability in microcompartment size (**c,d**) and silk protein storage.*

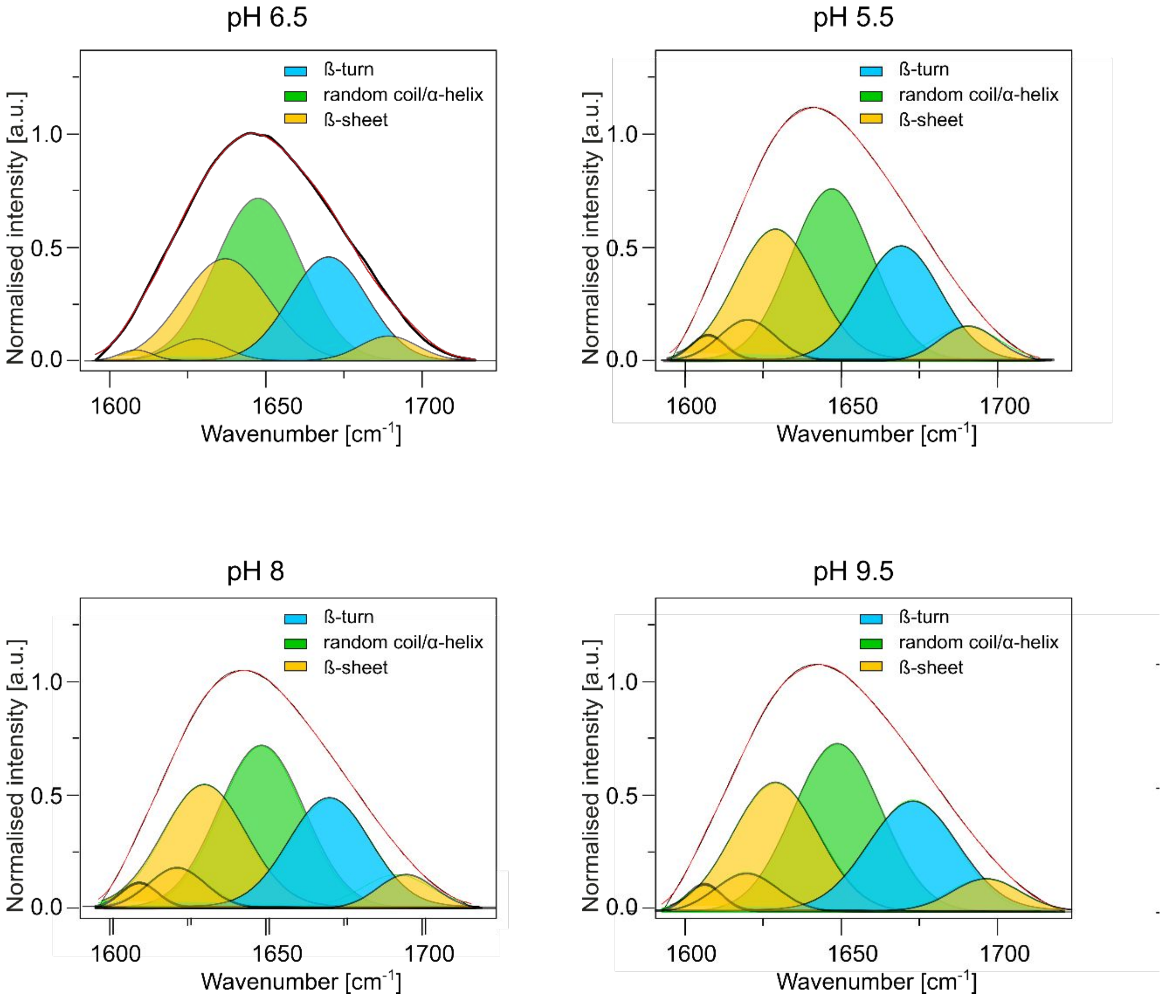

Supplementary Figure S4. *FTIR normalised absorption spectra in the range of the Amide I band (1595 – 1721 cm^{-1}) after solvent subtraction for all the RSF solutions (pH 5.5, 6.5, 8 and 9.5). The second derivative was calculated and deconvoluted by selecting the seven Gaussian peaks at the following wavelengths corresponding to the vibrational wavenumbers: 1609, 1621, 1631, 1650, 1695 and 1703 cm^{-1}.*[51–53] *The seven Gaussians approximate the absorption spectra for convergences having a chi-square tolerance value $< 1E^{-6}$.*

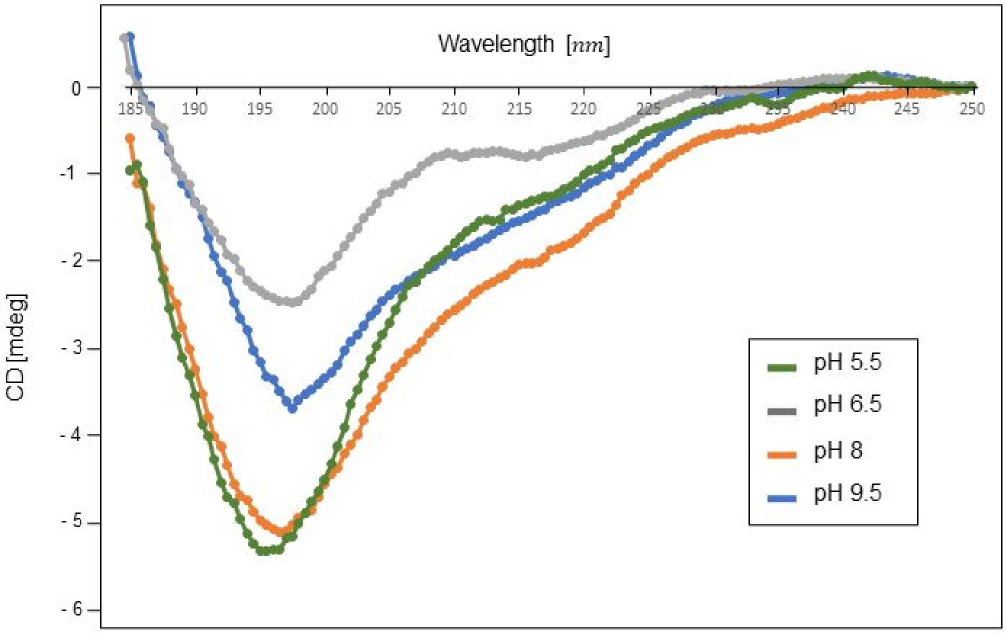

Supplementary Figure S5 *Circular Dichroism (CD) raw spectra collected for all the RSF solutions (pH 5.5, 6.5, 8 and 9.5) in the range of 185-250 nm using a J-715 spectropolarimeter having a data resolution of 0.5 nm. The RSF solutions were diluted until a concentration of 0.01 mg/ml and a 0.1 mm quartz cuvette were used.*

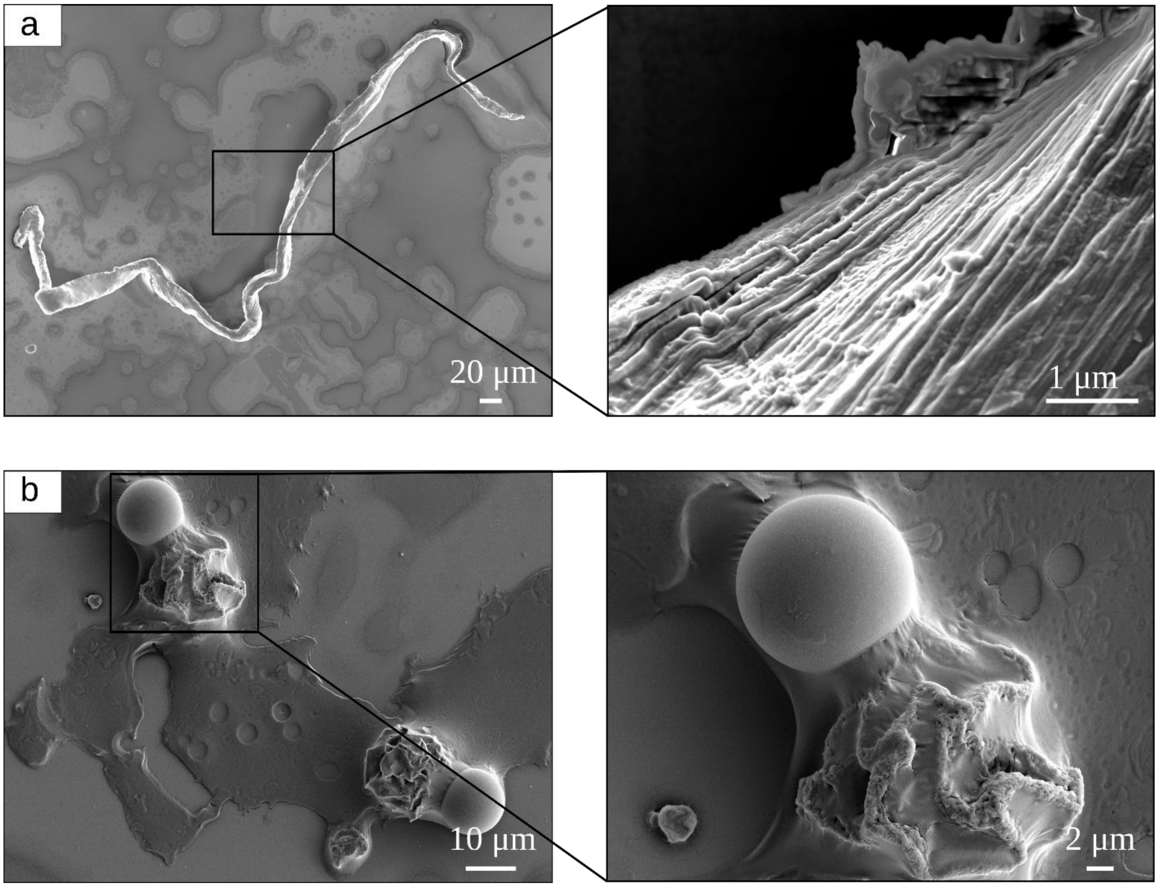

Supplementary Figure S6. *SEM microscopy for microcompartmentalised silk fibroin having environmental and fluidic conditions of pH 5.5, 7% concentration and Ca 0.1, respectively. The picture shows two of the myriad possible mechanisms of microcompartment generation.* ***a)*** *On the left, a macroscopic picture of a macroscopic assembled fiber, on the right a detail of the fibrils' distribution packaged together.* ***b)*** *Microcompartments with complex asymmetrical shapes, where two interconnected spheres or cylindrical compartments are linked, demonstrating different assembly levels.*

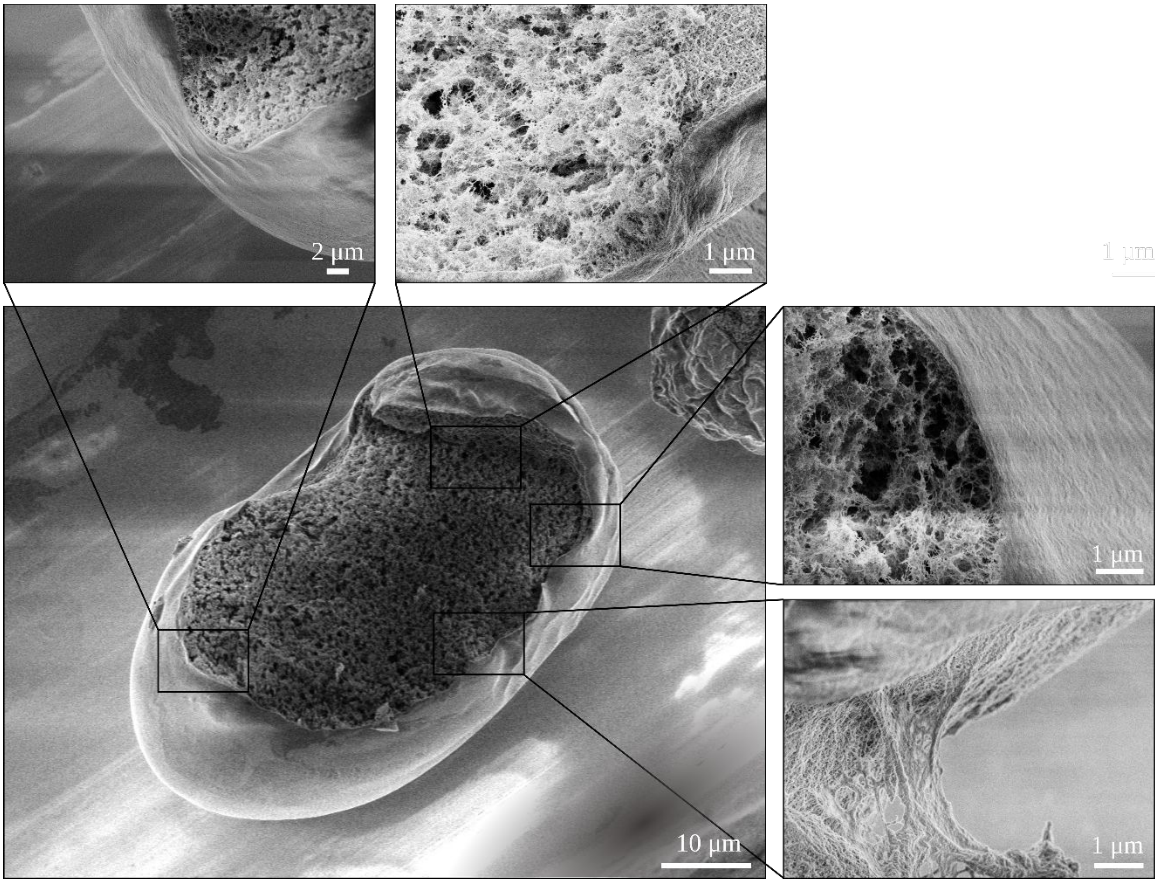

Supplementary Figure S7. *Cryo-SEM microscopy for microcompartmentalised silk fibroin having environmental and fluidic conditions of pH 5.5, 7% concentration and Ca 0.1, respectively. The pictures show the difference between the different aggregative states at the interface with respect to the bulk. The magnifications depict the higher level of aggregation of the microcompartment pulp compared with those having a higher pH value and a more pronounced protein assembly recurring at the outer shell.*

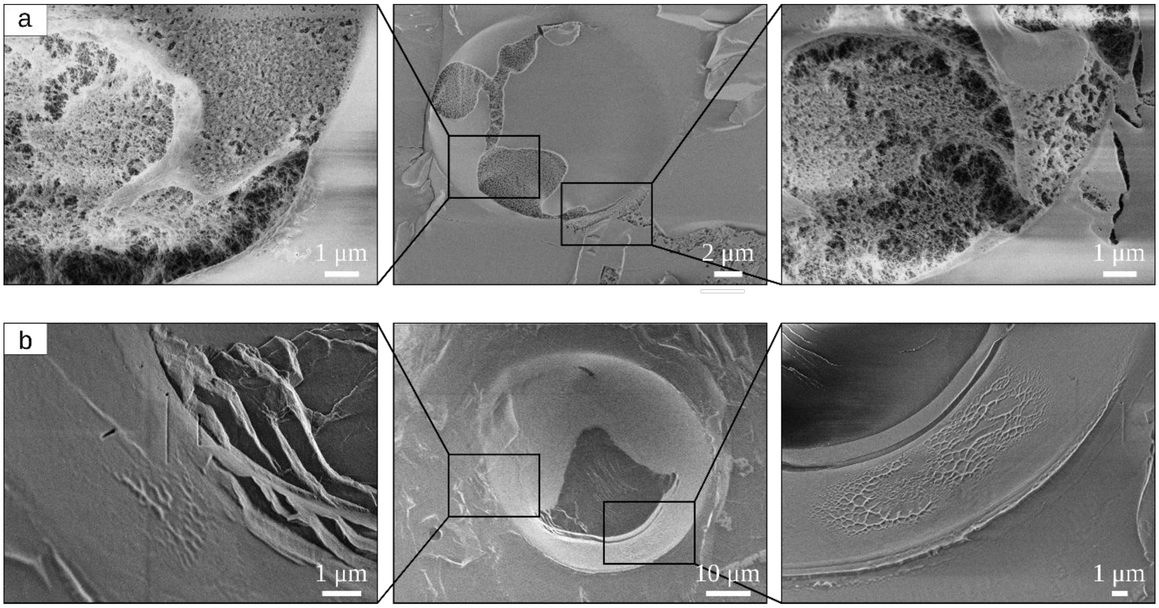

Supplementary Figure S8. *Cryo-SEM microscopy for microcompartmentalised silk fibroin having environmental and fluidic conditions of pH 8, 7% concentration and Ca 0.1, respectively. The pictures show the difference between the different aggregative states at the interface with respect to the bulk. The magnifications (left and right pictures) remark the morphological configuration of the microcompartment pulp (**a**) and the interface (**b**).*

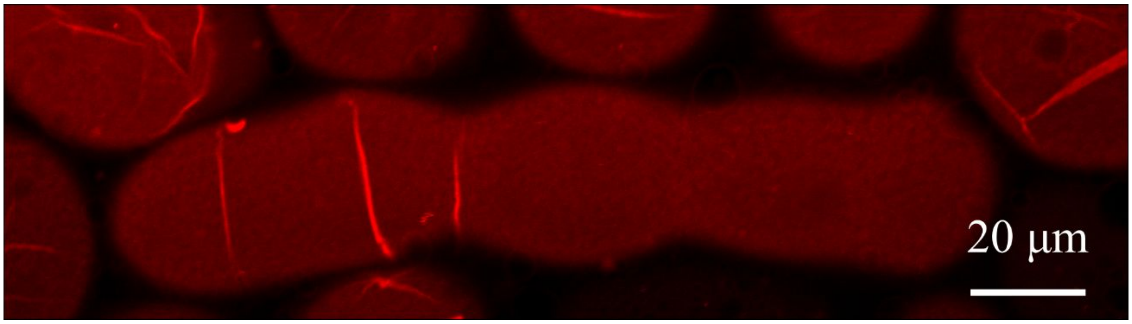

Supplementary Figure S9. *Optical fluorescent microscopy for microcompartmentalised silk fibroin having environmental and fluidic conditions of pH 5.5, a 7% concentration of fibroin, and Ca 0.1, respectively. The staining was performed using Nile Red.*

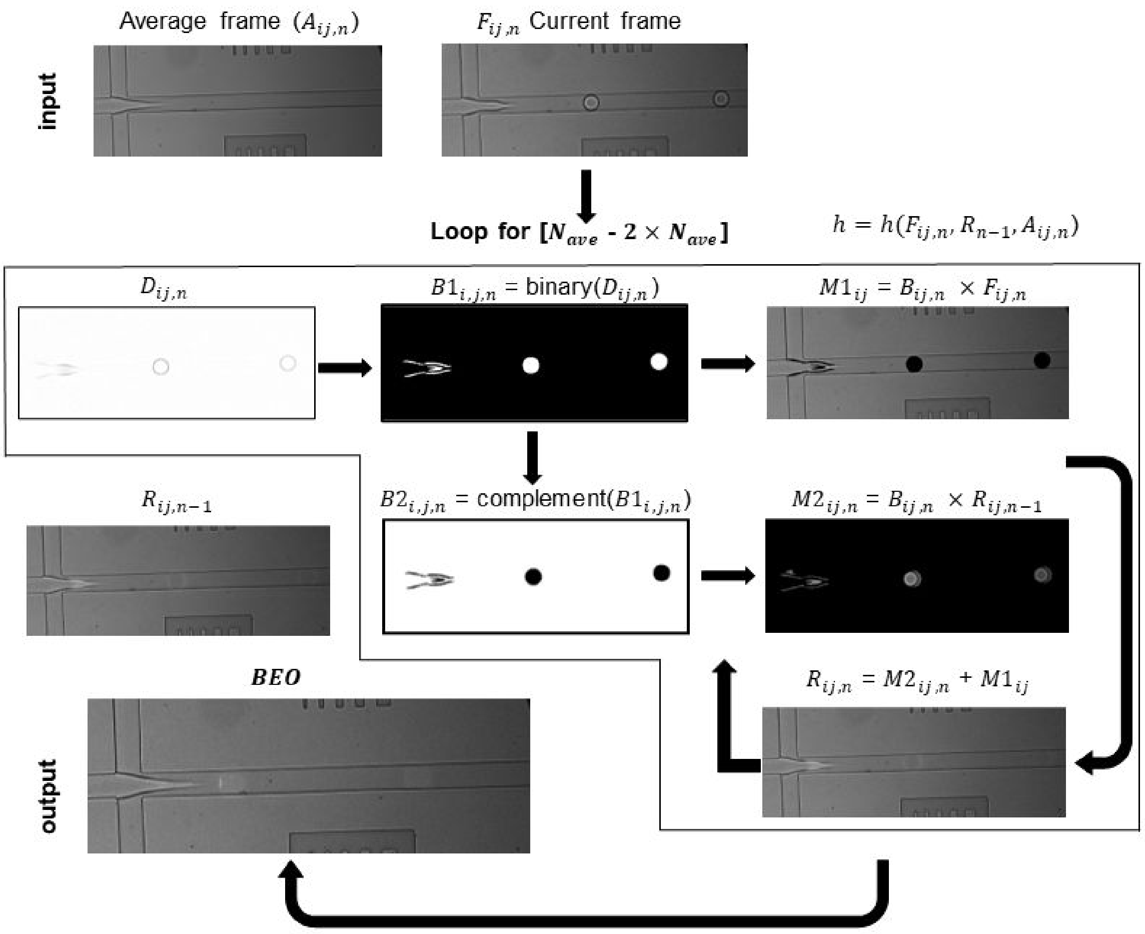

Supplementary Figure S10. *The Automated Droplet Recognition (ADR) algorithm flow chart leads to the Background Extraction Operation (BEO), to remove or attenuate any light interference or focal plane non-alignment. First, an average matrix of at least* ***N****=50 consecutive frames to obtain* $A_{ij,n}$ *is performed. Next, the loop is executed for the frame* $\boldsymbol{N}$, *until the frame* $\boldsymbol{N} \times 2$ *and using a BPR filter, described in the text, the* $D_{ij,n}$ *matrix is readily calculated. Then, the binary* $B1_{ij,n}$ *and the complementary* $B2_{ij,n}$ *matrix are multiplied by* $F_{ij,n}$ *(frame at time n) and* $R_{ij,n-1}$ *the (frame resulting from the loop at time n-1), respectively, to obtain* $M1_{ij,n}$ *and* $M2_{ij,n}$. *Eventually, the sum of* $M1_{ij,n}$ *and* $M2_{ij,n}$ *gives the partial result of* $R_{ij,n}$ *at time n. Recursively applying the loop for the subsequent* $\boldsymbol{N} \times 2$ *leads to the BEO matrix.*

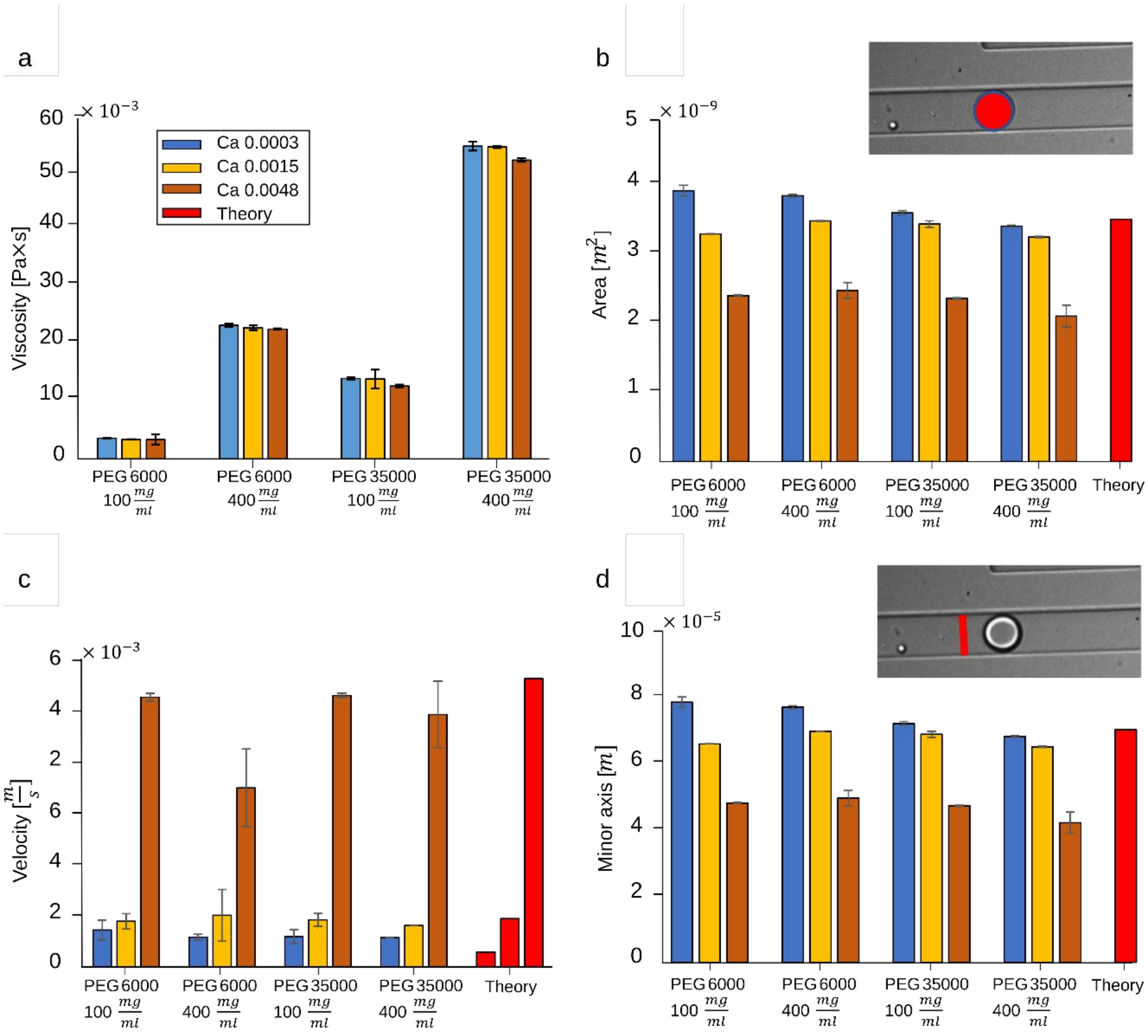

Supplementary Figure S11. ***a)*** *Dynamic viscosity measurements for four different solutions: PEG 6000KDa at a concentration of 100 [$\frac{mg}{ml}$] and 400 [$\frac{mg}{ml}$] and PEG 35000 KDa at a concentration of 100 [$\frac{mg}{ml}$] and 400 [$\frac{mg}{ml}$] at three different shear rates, thus having a Ca = 0.003, 0.015, 0.048, corresponding to squeezing, transition and dripping mode in a microfluidic regime.* ***b)*** *Automated area calculation for the four solutions at the three Ca numbers. In red, the theoretical dimensional area is presented and has a diameter equal to the characteristic channel dimension. Note that the dimensionalities exhibited an inverse correlation with the dynamic viscosity under the same fluid dynamic conditions.* ***c)*** *Experimental droplet velocity estimation knowing the fps and the pixel size, compared to the theoretical value. Importantly, the theoretical values define the fluid velocity without considering the mass of the droplets and thus explain the lower velocity.* ***d)*** *Minor axis estimation with ADR. These values demonstrate the reliability of droplet dimensionalities from the known channel width. Indeed, except for the case of Ca = 0.1, where the droplet is smaller than the channel width, the higher minor-axis value slightly overestimates the theoretical one.*

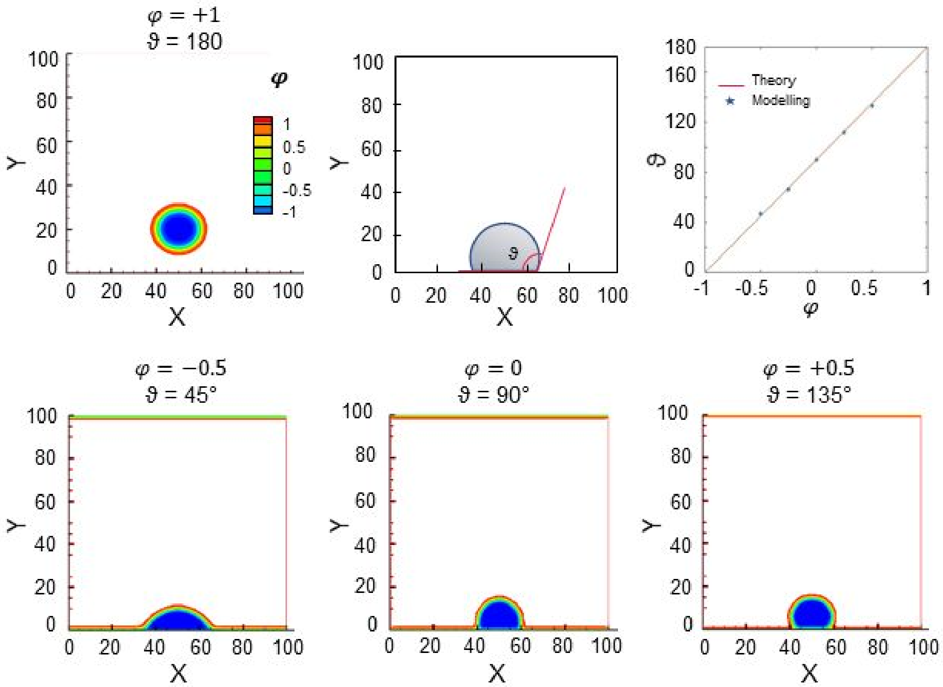

Supplementary Figure S12. *Computational results of droplet wettability on a flat surface (bottom). Initial conditions were set by placing half droplets directly in contact with the wall surface. (First row from left to right) three different imposed boundary conditions having* $\varphi = -0.5, 0, 0.5$ *defining, from eq.24, three different contact angles* $\vartheta = 45°, 90°$ *and* $135°$*, respectively. (Second row) full hydrophobicity was imposed by applying* $\varphi = 1$ *and causing a detachment. The graph shows the linear correlation between the wall* φ *and* ϑ *as expected from eq.24. The line represents the theoretical behaviour, whereas the dots represent the simulated results.*

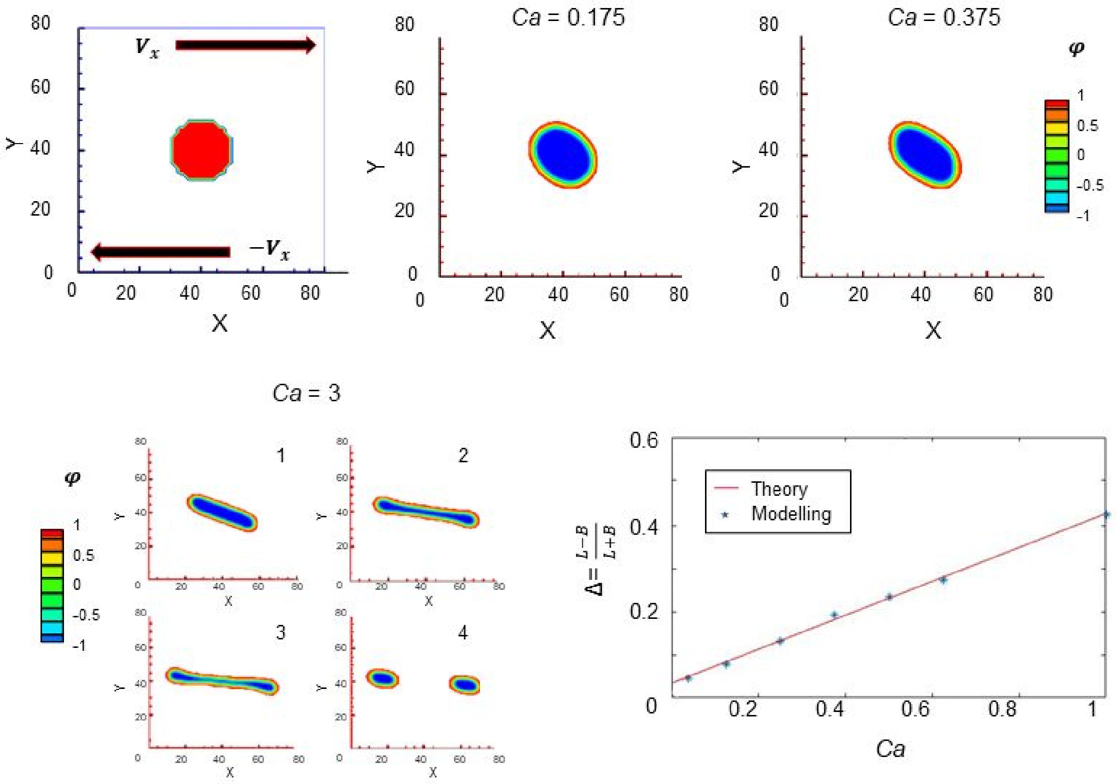

Supplementary Figure S13. *Simulated Taylor deformation under shear flow. The top and bottom boundaries have an imposed and opposite velocity inducing only shear flow to the object. (First row from left to right) the initial conditions of a discrete droplet placed in the centre of the computational domain; periodic boundary conditions were imposed in the x-direction. Droplet deformation under* $Ca = 0.175$ *and* 0.375. *(Second row) rheology of a droplet breakage estimated for Ca = 3. As confirmed, for low Ca numbers, the process is linear.*

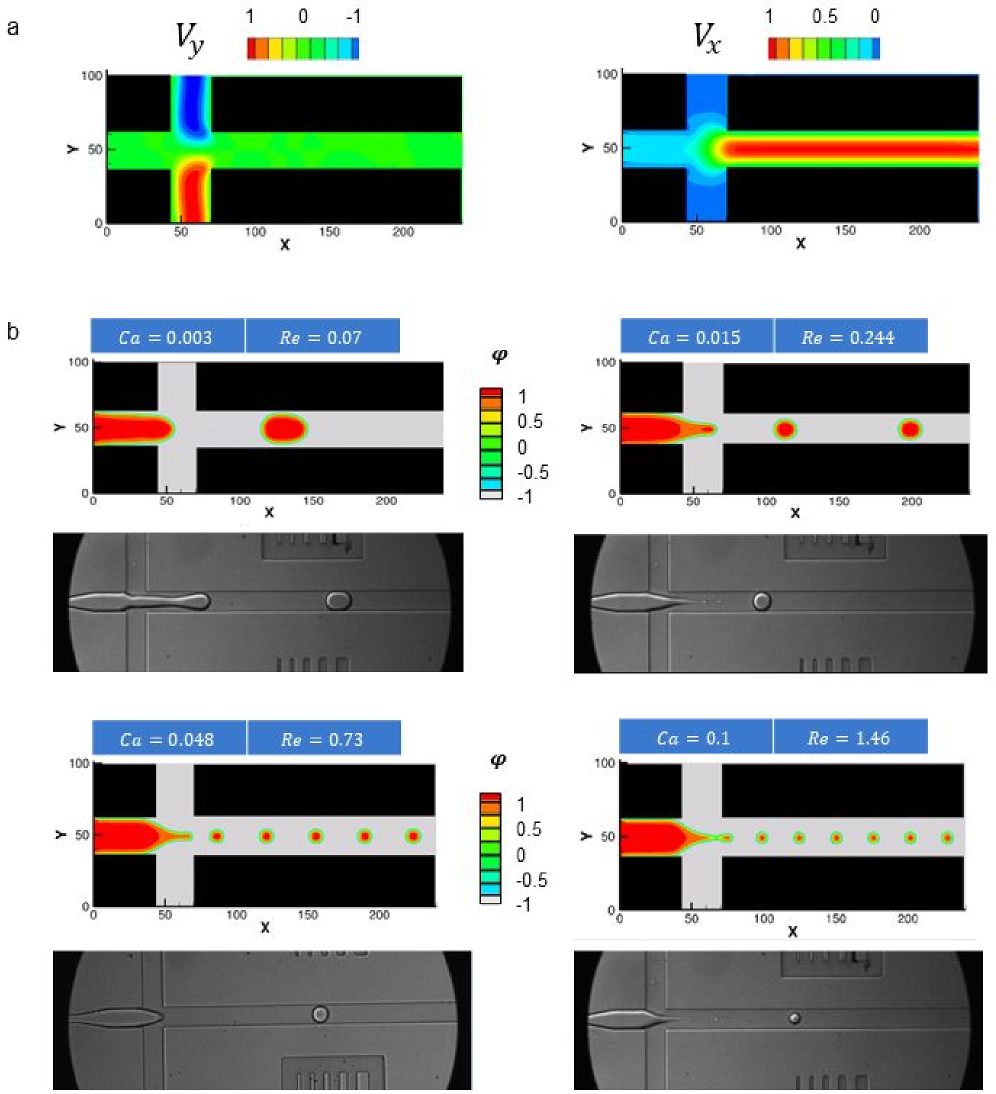

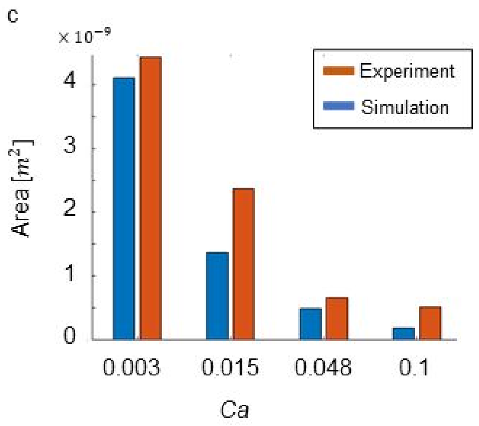

Ca	0.003	0.015	0.048	0.1
Re	0.06	0.245	0.73	1.4

Supplementary Figure S14. *Simulation results of binary fluids, modelling the droplet rheology in a droplet maker microfluidic chip having a cross-section. Mechanical material properties and fluid*

dynamic conditions with respect to the experimental parameters. ***a)*** *The distribution of the non-dimensional fluid velocity components,* V_x *and* V_y*, at the cross-section.* ***b)*** *Four different simulations replicating the four Ca and the Re number imposed experimentally. The pictures depict the differences between droplet dimensionalities from the experiment and simulation.* ***c)*** *Bar graph resuming the experimental and simulated area of the droplet for four fluid dynamic conditions. Note that to calculate the experimental area, the ADR algorithm was used, whereas for the modelled droplet, the contour that identifies* $\varphi = 0$ *defines the interface of the droplet. Note that given the continuity of the Ginzburg-Landau free energy function, the ordered parameter* φ *variation expects an interface thickness from the positive value (the fluid) to the negative value (the droplet). This leads to a slight diameter reduction, and thus the area of the simulated droplets underestimates the actual dimensionalities.*

References

(1) Chong, Z. Z.; Tor, S. B.; Gañán-Calvo, A. M.; Chong, Z. J.; Loh, N. H.; Nguyen, N. T.; Tan, S. H. Automated Droplet Measurement (ADM): An Enhanced Video Processing Software for Rapid Droplet Measurements. *Microfluid. Nanofluidics* **2016**, *20* (4), 1–14. https://doi.org/10.1007/s10404-016-1722-5.

(2) Li, Y.; Ward, K. R.; Burns, M. A. Viscosity Measurements Using Microfluidic Droplet Length. *Anal. Chem.* **2017**, *89* (7), 3996–4006. https://doi.org/10.1021/acs.analchem.6b04563.

(3) Liu, H.; Zhang, Y. Droplet Formation in a T-Shaped Microfluidic Junction. *J. Appl. Phys.* **2009**, *106* (3). https://doi.org/10.1063/1.3187831.

(4) Rockwood, D. N.; Preda, R. C.; Yücel, T.; Wang, X.; Lovett, M. L.; Kaplan, D. L. Materials Fabrication from Bombyx Mori Silk Fibroin. *Nat. Protoc.* **2011**, *6* (10), 1612–1631. https://doi.org/10.1038/nprot.2011.379.
